# Aluminum enhances the oxidative damage of ZnO NMs in the human neuroblastoma SH-SY5Y cell line

**DOI:** 10.1186/s11671-024-03973-2

**Published:** 2024-02-26

**Authors:** Arturo Jimenez-Chavez, Gladis Pedroza-Herrera, Israel Betancourt-Reyes, Andrea De Vizcaya Ruiz, David Masuoka-Ito, Juan Antonio Zapien, Iliana E. Medina-Ramirez

**Affiliations:** 1 Departamento de Toxicología, Centro de Investigación y de Estudios Avanzados de IPN (CINVESTAV-IPN), Ciudad de Mexico, México; 2 Department of Chemistry, Universidad Autónoma de Aguascalientes, Av. Universidad 940, Aguascalientes, Ags Mexico; 3 Instituto de Investigaciones en Materiales, Universidad Nacional Autonoma de México, Mexico, México; 4 Department of Stomatology, Universidad Autónoma de Aguascalientes. Av. Universidad 940, Aguascalientes, Ags Mexico; 5 Department of Materials Science and Engineering, City University of Hong Kong, Hong Kong SAR, People’s Republic of China; 6 Department of Environmental and Occupational Health, Program in Public Health, Susan and Henry Samueli College of Health Sciences, University of California Irvine, Irvine, CA USA

## Abstract

Bare and doped zinc oxide nanomaterials (ZnO NMs) are of great interest as multifunctional platforms for biomedical applications. In this study, we systematically investigate the physicochemical properties of Aluminum doped ZnO (AZO) and its bio-interactions with neuroblastoma (SH-SY5Y) and red blood (RBCs) cells. We provide a comprehensive chemical and structural characterization of the NMs. We also evaluated the biocompatibility of AZO NMs using traditional toxicity assays and advanced microscopy techniques. The toxicity of AZO NMs towards SH-SY5Y cells, decreases as a function of Al doping but is higher than the toxicity of ZnO NMs. Our results show that N-acetyl cysteine protects SH-SY5Y cells against reactive oxygen species toxicity induced by AZO NMs. ZnO and AZO NMs do not exert hemolysis in human RBCs at the doses that cause toxicity (IC50) in neuroblastoma cells. The Atomic force microscopy qualitative analysis of the interaction of SH-SY5Y cells with AZO NMs shows evidence that the affinity of the materials with the cells results in morphology changes and diminished interactions between neighboring cells. The holotomographic microscopy analysis demonstrates NMs' internalization in SH-SY5Y cells, changes in their chemical composition, and the role of lipid droplets in the clearance of toxicants.

**Graphical Abstract:**

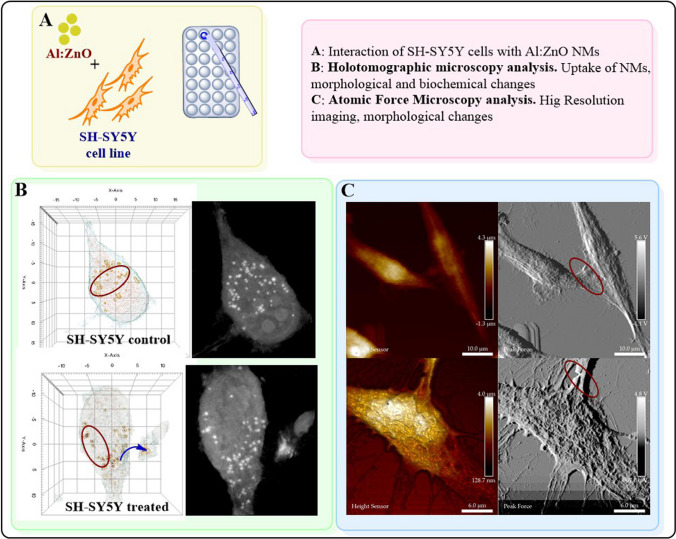

**Supplementary Information:**

The online version contains supplementary material available at 10.1186/s11671-024-03973-2.

## Introduction

Chronic diseases (CDs) such as cancer, diabetes, cardiovascular and neurodegenerative diseases are increasing in number and magnitude due to changes in the population's mean age average, healthy habits, and environmental pollution [1]. The diseases related to the brain are a big challenge for the scientific community; thus, there is a need to do more research to understand their mechanisms and treatment [2]. Nowadays, cancer is the second leading cause of death in the United States [3]. Although many strategies exist for cancer treatment, conventional therapies result in side effects on patients (damage to healthy cells) and the development of resistance in cancer cells after exposure to repeated treatment. Lately, numerous studies support the efficiency of nanomedicines for cancer management; in particular, ZnO (zinc oxide) exhibits specificity in its bioactivity against cancerous cells [4–10].

Currently, several nanomedicines find application in the treatment and diagnosis of different diseases; however, it is still necessary to develop NMs highly biocompatible, cheap, biodegradable, and simple to fabricate. In this regard, ZnO fulfills the requirements; in addition, because of its optical and redox properties, this material is suitable for numerous biomedical applications [11–13]. For example, a recent review highlights the advantages of using ZnO for cancer treatment due to the specific cytotoxic activity of ZnO NPs on cancer cells by reactive oxygen species (ROS) generation and mitochondrial membrane potential destruction. This study also points out the suitability of the material as an anti-microbial, anti-diabetic, anti-inflammatory, wound healing, and bio-imaging agent [10]. Furthermore, the Food and Drug Administration (FDA) classifies ZnO as a "GRAS" (generally regarded as safe), which facilitates its biomedical applications. Not surprisingly, extensive research efforts aim to fabricate ZnO materials with optimal properties for diverse therapeutic uses [14, 15].

The doping of ZnO with main group elements provides materials with improved optoelectronic properties, bioactivity, and diverse morphologies. For example, aluminum-doped zinc oxide (AZO) is of commercial interest because of its similarity to Indium Tin Oxide (ITO) but at a lower cost and reduced toxicity [16]. Also, recent reports highlight the enhanced and selective cytotoxic activity of AZO materials in MCF-7 cells [17]. Oxidative stress is the principal mechanism to induce apoptosis in cancer cells by treatment with ZnO NMs. Previous studies demonstrated that active redox species (electrons and holes; e^−^/h^+^) are present in ZnO NMs even without UV light activation [18]. Furthermore, Al doping increases the production of e^−^/h^+^ pairs, enhancing the oxidative capacity of the AZO materials. Currently, extensive research efforts seek to tune the properties of AZO NMs by achieving a systematic control in dopant incorporation, size, shape, and reproducibility in AZO fabrication [19].

The electronic properties of AZO depend on the incorporation of dopants in substitutional sites of the ZnO matrix. There are different approaches for the synthesis of AZO, such as solvothermal [20], sol–gel [21], precipitation [22], gas condensation [23], hydrothermal process [24], and hydrolysis in polyol medium [25]. Among them, the synthesis of AZO materials using a microwave-activated solvothermal approach allows the fabrication of the crystalline materials within minutes and facilitates the optimization of the synthesis variables. We are not only interested in developing NMs with optimal properties for practical applications but are also concerned about their biocompatibility.

Amidst this frenzy of application-driven research, we join the ranks of those that increasingly call for the need to complete toxicity studies that must be conducted before commercialization [26–28]. Failure to perform such toxicity studies can lead to unintended risks; derived from the use of nanomaterials. As is the case, for example, of the extensive use of quantum dots (CdSe) as bio-imaging agents while these materials show hepatocyte toxicity due to the leakage of Cd^2+^ ions [29]. Aluminum is a non-oxidizing metal; however, lately, the pro-oxidant activity of this metal is associated with several neurodegenerative diseases [30]. Although numerous studies claim the non-toxic nature of AZO materials, it is imperative to understand the extent and means of interactions between them and living cells.

In this study, we investigated the changes in the physicochemical properties of AZO as a function of doping level. We also study the bio-interaction of AZO NMs with SH-SY5Y and RBCs cells. We previously highlighted the importance of Atomic Force Microscopy (AFM) analysis to understand NMs-cell interactions; however, AFM has limitations upon NM uptake and its bio-distribution [13]. Thus, this study uses Holotomographic Microscopy (HTM) as a complementary technique to AFM to investigate the AZO-cell interactions. HTM is an emerging technique with an efficient resolution that requires minimal sample preparation and performs quantitative and cellular localization of NMs at the single-cell level. In summary: a) this work provides a reliable synthetic pathway to prepare AZO nanocrystalline materials; b) It also demonstrates changes in the physicochemical properties and biocompatibility of NMs which are dopant dependent; c) Lastly, the use of AFM and HTM to study NMs-cell interactions supports and clarify data generated by classical toxicity assays.

## Experimental

### Synthesis of AZO NMs

We previously described in detail the protocol for the synthesis of NMs [31]. In brief, we mix proper amounts of the reactants (zinc acetate dihydrate, aluminum nitrate, and benzyl alcohol). Then the mixture undergoes thermal treatment (first, heating at 60 °C for 2 min, then heating at 180 °C for 3 min). Finally, the purification of the NMs by washing three times with ethyl ether. The powders are separated by centrifugation and dried at 70 °C for 12 h.

### Characterization of AZO NMs

The NMs were fully characterized using several advanced procedures. The size and shape of the NMs were investigated by Scanning Electron Microscopy analysis (SEM, JSM-7600F, JEOL with a secondary electron detector). X-ray diffraction, XRD (SIEMENS D-500 VPCEA4OEL) is used to determine the crystallinity and crystallite size of the NMs. Applying the Scherrer equation, we calculate the crystallite size from the Full Width Half Maximum (FWHM) of the most intense reflection (101). The morphology of ZnO and AZO (1, 3 and 5 at. % Al/Zn) was analyzed by High-Resolution Transmission Electron Microscopy (HRTEM) using a JEOL ARM 200F (Tokyo, Japan) operating at 200 kV. Analysis of selected area diffraction (SAED) patterns of the NMs, denotes their crystalline phases.

The optical properties (band gap) of ZnO and AZO NMs, were studied by UV–Vis diffuse reflectance spectroscopy (Shimadzu UV-2600i). The purity and chemical composition of the NMs were investigated by XPS analysis (SPEC Phoibos 150 equipment operated at 10 mbar and provided with a monochromatized Al Ka X-ray source (1487 eV). Monitoring the position of the O1s peak at 530.0 eV on each sample ensures that no binding energy shift due to charging had occurred. Narrow scans were collected at 60 eV analyzer pass energy and a 400 mm spot size. Charging effects were minimized by depositing the samples over an indium film [12].

### Cell culture

Chronic exposure to aluminum has been linked to neurodegenerative disorders. We use SH-SY5Y cells to investigate their biological response to the NMs under study and the cytotoxicity of AZO NMs against them. The human neuroblastoma SH-SY5Y cell line was obtained from ATCC (CRL-2266™). They were grown in Dulbecco´s Modified Eagle´s Medium (DMEM) medium supplemented with 1% (v/v) penicillin, 1% (v/v) streptomycin, 1% (v/v) pyruvate, 1% (v/v) glutamine, 1% non-essential amino acids and 10% (v/v) inactivated FBS. The cells were kept at 37ºC in a humidified atmosphere with 5% CO_2_. For toxicity studies, the cells were seeded in 96 well plates at 5 × 10^4^ cells/cm^2^ concentration and allowed to adhere for 48 h before NMs exposure.

### NMs suspension

Stock solutions (1 mg/mL) of AZO NPs with different doping (1%, 3%, 5% at.% Al/Zn) were prepared in double-distilled water and sonicated for 3 min in an ice bath (40% of amplitude in a tip sonicator Q125, Qsonica, USA). After suspension, the NMs were diluted at different concentrations (µg/mL) in 1% FBS DMEM cell culture medium.

#### Physicochemical characterization of NMs suspensions

We evaluate the stability of AZO NPs at different Al doping concentrations (1%, 3%, 5% at. % Al/Zn) in water or culture medium under several conditions: a) Variable sonication time and sonication method (tip or bath sonication); b) In the same manner, we determine the stability of NPs suspension (100 µg/mL in DMEM medium supplemented with 1% FBS) at 0, 24, and 48 h. We determined the hydrodynamic diameter of NPs by dynamic light scattering analysis (DLS) and the Zeta potential by Laser Doppler microelectrophoresis (Zetasizer NanoZS90 de Malvern Instruments Ltd, UK.). The NMs' effective density in water and in DMEM medium was determined by the protocol described by DeLoid et al., 2014. Briefly, a suspension of 1 mg/mL of each AZO NM in DMEM medium with 1% FBS or water was prepared; 1 mL of the suspension was placed in a packed cell volume (PCV) tube and centrifuged by 1 h at 3,000 g. Past this time the formed NMs pellet in the PCV tube was measured in the “easy read” measure device and effective density was calculated according to the DeLoid et al., 2014 formula [33].

### Cell viability and inhibitory concentration 50 calculation

After 24 h of exposure to AZO NMs, we performed two different cell viability techniques, the LDH and MTS assays. We measured the release of LDH to cell media according to the manufacturer protocol (LDH Cytotoxicity detection kit Roche, catalog no. 11644793001). For the MTS assay, after 24 h of exposure to AZO NMs, the cells were washed twice with PBS solution. 100 µL of MTS solution (1:10) in DMEM medium was added to each well and incubated for 2.5 h to obtain the soluble formazan salt. Following this period, the medium with the formed formazan salt was removed and taken to absorbance reading at 490 nm for MTS in a TECAN Infinite® 200 spectrophotometer. The inhibitory concentration 50 (IC50) was determined for each NM by interpolation of the results from the MTS assay. We also investigate the possible contribution of oxidative damage as an adverse effect due to exposure to AZO NMs. The cells were pre-exposed to N-acetyl-cysteine (NAC). Under the same conditions, SH-SY5Y cells were pre-exposed for one h to 10 mM solution of NAC before 24 h co-exposure with or without AZO NMs.

### Analysis of the hemocompatibility of ZnO and AZO NMs

We evaluate the hemocompatibility of the NMs under study by exposing RBCs (whole blood) to the different NMs using their IC50 encountered for SH-SY5Y cells. Three blood samples came from three healthy donors (no chronic diseases diagnosed, 20–30 years old). Donors attended the sample collection with a minimum fasting of 6 h. We evaluate the hemolytic potential of the materials by the ASTM F756-13 standard (Standard practice for assessment of hemolytic properties of the materials). Briefly, Heparin-stabilized human blood was freshly collected. After, we added physiological saline solution (PSS) to test tubes (10 mL of PSS in each tube). Then, addition of the NMs to each tube according to their IC50 values (Table 1). Finally, we added 100 µL of whole blood to each tube. Tubes were incubated at 37 °C in a water bath at 65 RPM for three hours. After incubation, tubes were centrifugated at 3500 rpm for 5 min. We determine the amount of hemoglobin by UV–Vis spectroscopy (Helios Omega Thermo Scientific TM spectrophotometer).

**Table 1:** Determination of the hemocompatibility of ZnO and AZO NMs

Tube	NMs Concentration (µg/mL)			
	ZnO	AZO 1%*	AZO 3%**	AZO 5%**
Control (−)*				
1	80	15	21	26
2	90	25	31	36
3	100	35	41	46
Control ( +)**				

*9.9 mL PSS + 100 µL whole blood; **9.9 mL Distilled water + 100 µL whole blood. Aluminum doped zinc oxide (AZO) at different doping levels 1*, 3**and 5*** atomic % Al/Zn

We studied the interaction of RBCs with ZnO and AZO NMs using Holotomographic microscopy. HT microscopy was carried out with a sample of 10 µL, placed in a glass substrate, and covered with coverslips. The samples were analyzed with an HT-2L microscope (Tomocube Inc.).

### Analysis of the interaction between SH-SY5Y cells and ZnO doped NPs by atomic force microscopy (AFM)

The interaction of the NPs with the cell membrane, in particular morphological changes of SH-SY5Y cells membrane after interaction with AZO was investigated by AFM (Bruker dimension Edge AFM) using a silicon tip on nitride lever (70 kHz, scanasyst-air). All images, 256 × 256 pixels and line scan rate 0.300 Hz, were analyzed using NanoScope Analysis software.

The cells were seeded on coverslips and incubated for 48 h. A control group was separated and fixed, while the cells exposed to AZO NPs were allowed to interact for further 24 h using a 5 µg/mL solution of AZO NPs. After exposure, the media was removed, and cells were fixed in 10% buffered paraformaldehyde and dried with ethanol at increasing concentrations. Finally, analysis of all the cells (control and exposed groups) was performed using Atomic Force Microscopy to investigate morphology changes in the cells.

### Analysis of the interaction between SH-SY5Y cells and AZO NPs by Holotomographic Microscopy (HTM).

HTM is capable to perform qualitative analysis of live cells from bright-field images, 3D refractive index (RI) reconstruction of the cells, and three-channel 3D fluorescence imaging. It can also perform quantitative analysis of the live cells by measuring variations in lipid droplets content, cell volume, and morphology changes. More details on HTM imaging can be seen in Ref. [34]. We use a commercial HTM (Tomocube HT-2; Tomocube, Inc, /Daejeon, Korea) to perform both qualitative and quantitative studies of the interaction of AZO NMs with live SH-SY5Y cells.

SH-SY5Y cells were cultured in DMEM medium as previously described. Cells were seeded in HTM plates and allowed to adhere 48 h before NMs exposure whereas the control group was not exposed to NMs but were allowed to incubate for further 24 h. Cells were exposed to 5 µg/mL of AZO NPs for 24 h. 3D images of the cells are reconstructed using HTM’s software. To interpret the 3D RI tomograms, the cell components and NMs are segmented by thresholding RI values, using their proper RI values between cell membrane (1.3450–1.3506; blue color), cytoplasm (1.3749–1.3824; pink color), lipid droplet (1.3800–1.3900; yellow) and NMs (> 1.4019; red color).

### Statistical analysis

We analyze the data obtained from the SH-SY5Y cell viability test by a one-way ANOVA test followed by the Bonferroni post hoc test. We used the Kruskal–Wallis followed by Dunn´s post hoc test to analyze the data from the hemocompatibility and the quantitative characterization of RBCs (HTM). Data were considered significant at *p < 0.05 or **p < 0.001. Statistical software Prism 8.0 (GraphPad Software, Inc. US).

## Results and discussion

### Synthesis of AZO NMs

**Fig. 1:**
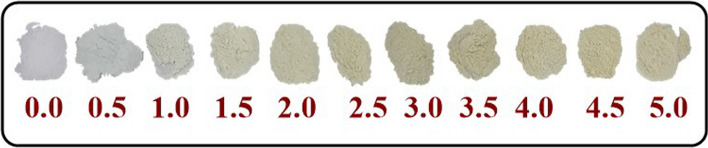
Schematic representation of bare and doped ZnO NMs

In this study, we present a facile and robust strategy for synthesizing NMs of bare and doped ZnO (AZO) within minutes (Eqs. 1 and 2). The easiness of the method and the kinetics of the reaction allow for a systematic and detailed study on the effect of dopant and dopant level on the physicochemical properties of ZnO, Fig. 1 shows the physical appearance of the powders as a function of increasing Al^3+^doping. AZO materials have a creamy color that do not change drastically when increasing the amount of Al^3+^ doping.

1 $$Zn\left( {Ac} \right)_{2} \mathop \to \limits^ {\begin{array}{*{20}c} {Benzyl alcohol} \\ {MW} \\ \end{array} }ZnO$$

2 $$Zn\left( {Ac} \right)_{2} + Al\left( {NO_{3} } \right)_{3} \mathop \to \limits^{{\begin{array}{*{20}c} {Benzyl~alcohol} \\ {MW} \\ \end{array} }} ZnO - Al^{{3 + }}$$

### Characterization of ZnO and AZO NMs

#### Scanning electron microscopy (SEM.EDX)

**Fig. 2:**
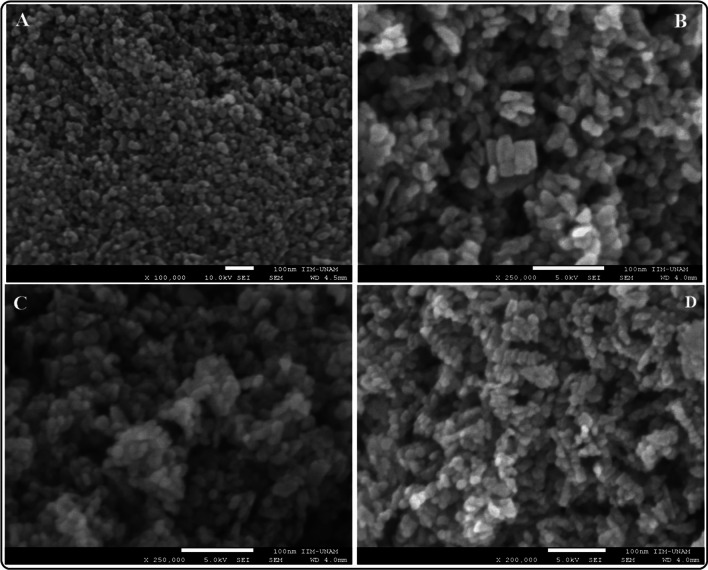
Low-resolution SEM micrographs of ZnO (**A**) and AZO NMs (1.0, 3.0 and 5 at. % Al/Zn) (**B**–**D)**

Figures 2 and Fig. 2S show a low-resolution SEM micrograph of ZnO and AZO powders. The NMs possess a small regular spherical shape. The size of the materials decreases slightly as the doping concentration increases. The reduced size of AZO materials is due to compression stress and the differences in the size of metal ions (Zn^2+^  = 0.074 nm, Al^3+^  = 0.053 nm); this size decrease supports the insertion of Al^3+^ions into the ZnO lattice [35]. EDX analysis denotes the incorporation of Al^3+^ into the ZnO lattice. The amount of aluminum doping in the materials is low, and the uncertainty in the measurement increases as the amount of doping is expected to increase (according to the experimental synthesis procedure).

**Fig. 3:**
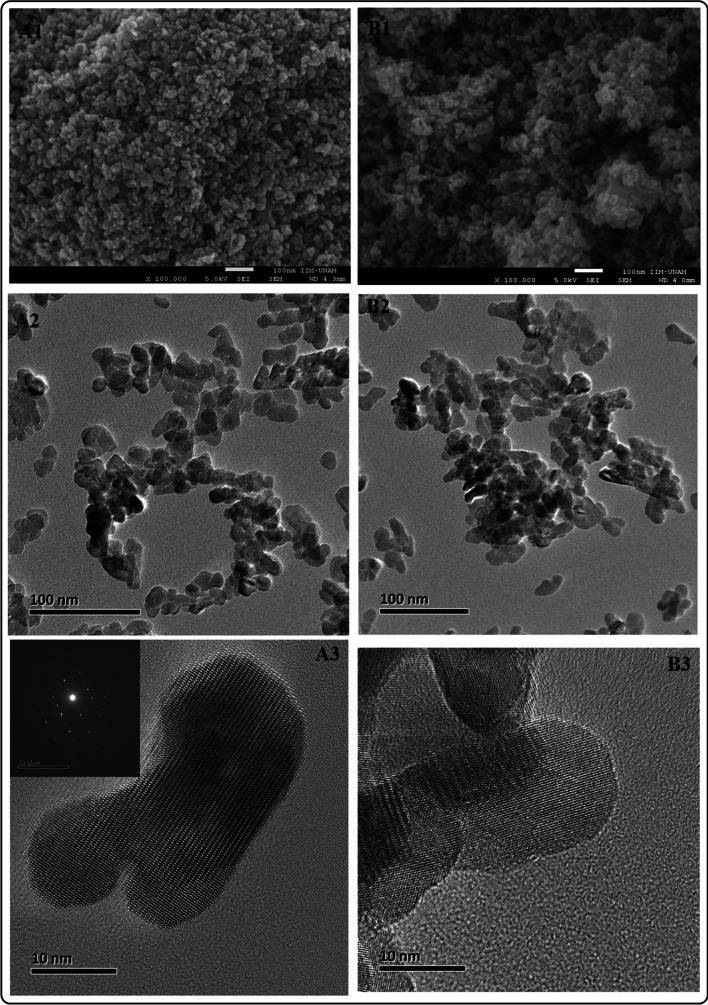
SEM and TEM analysis of AZO materials. **A1**–**A3** ZnO–Al 1%. **B1**–**B3** ZnO–Al–5%

Previous studies report a solubility limit of 0.5 at% of aluminum ion into the ZnO lattice. Our results indicate that using aluminum (III) nitrate as the aluminum precursor and benzyl alcohol as a solvent for the microwave-activated synthesis of the AZO materials, it is not possible to exceed the previously reported limit of solubility of aluminum ion due to the limited solubility of aluminum precursor in the solvent (Fig. 3S) and the mechanism of reaction to incorporate the ion into the ZnO lattice. Further studies will explore the feasibility of increasing the limit of solubility of aluminum ions by using precursors (aluminum acetates or acetylacetonate) that react with the solvent to facilitate the synthesis of ternary (Al_x_Zn_1-x_O) oxides.

Table 2 summarizes the physicochemical properties of ZnO and AZO NMs. We present a detailed characterization of ZnO NMs fabricated under the same conditions as AZO to remark on the differences in physicochemical properties due to the chemical nature of doping and doping level (Table 2, Fig. 1S).

**Table 2:** Physicochemical properties of ZnO and AZO NMs

Material	Size ± SD (SEM)	Elemental composition atomic (%)			Crystallite size (XRD)	Eg (eV)
		Zn	O	Al		
ZnO	18.45 ± 3.63	45.09 ± 2.15	54.91 ± 1.76		20.71 ± 0.73	
ZnO–Al^3+^ 1%	17.74 ± 2.37	46.45 ± 1.67	52.17 ± 1.34	1.37 ± 0.54	18.74 ± 0.33	3.009
ZnO–Al^3+^ 3%	16.05 ± 1.85	52.81 ± 1.98	46.28 ± 2.63	0.92 ± 1.01	17.06 ± 0.45	3.018
ZnO–Al^3+^ 5%	14.86 ± 1.31	52.95 ± 6.35	46.73 ± 6.15	0.33 ± 1.42	16.47 ± 0.29	3.047

A more detailed characterization of AZO materials by SEM, TEM, and HRTEM show that AZO NMs have different shapes and uniform sizes (Figs. 3A1, A2, B1, B2, 4S). HRTEM and selected area diffraction patterns (SAED) demonstrate a reduction in size dependent on the amount of doping and crystallinity of the materials. Figure 3A3 and B3 show highly crystalline particles with well-formed crystal lattices. The clear ring patterns indicate the formation of highly crystalline particles.

In a previous literature report, they evaluate the sensing properties of AZO materials for acetone. Fabricating the materials using two routes (hydrothermal and Spray Flame Pyrolysis) renders AZO NMs with different properties (including shape) and sensing capacities [36]. Further studies from our research group seek to evaluate other variables in the synthesis of AZO NMs (pressure, aluminum precursor), aiming to improve their activity and biocompatibility.

#### X-Ray diffraction (XRD)

**Fig. 4:**
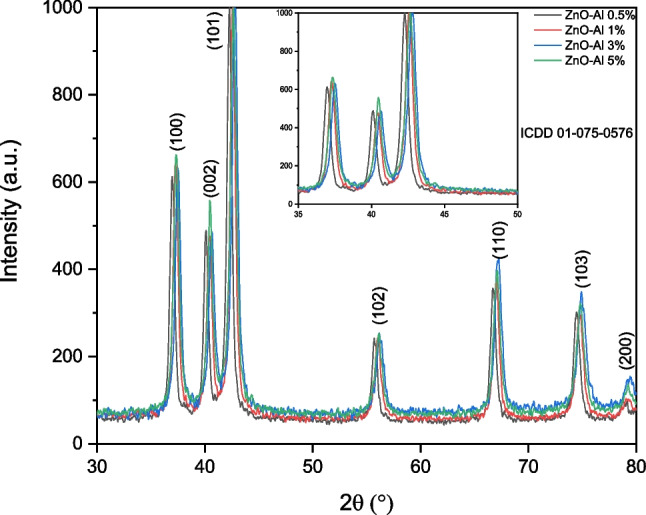
XRD analysis of AZO NMs

**Fig. 5:**
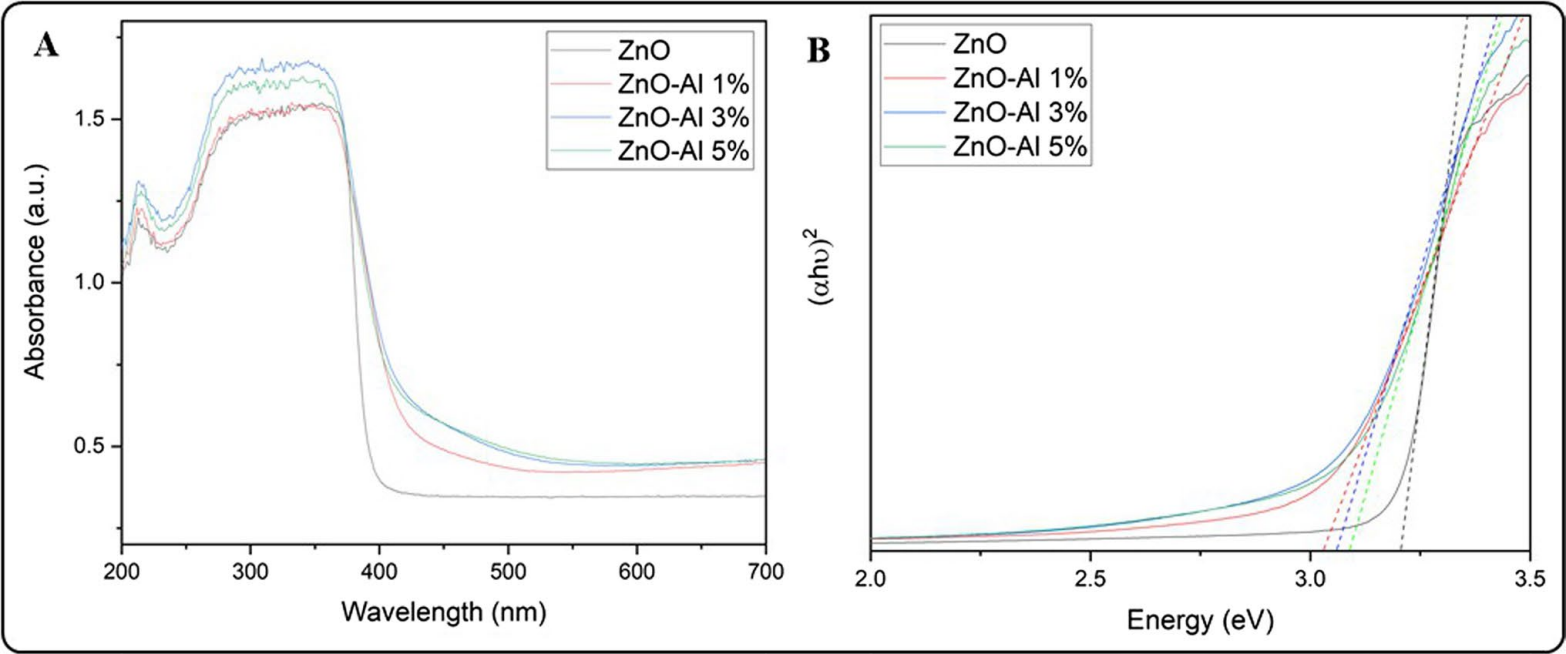
**A** UV–Vis absorption properties of AZO NMs. **B** Diffuse reflectance spectroscopy analysis of bare and AZO NMs

**Fig. 6:**
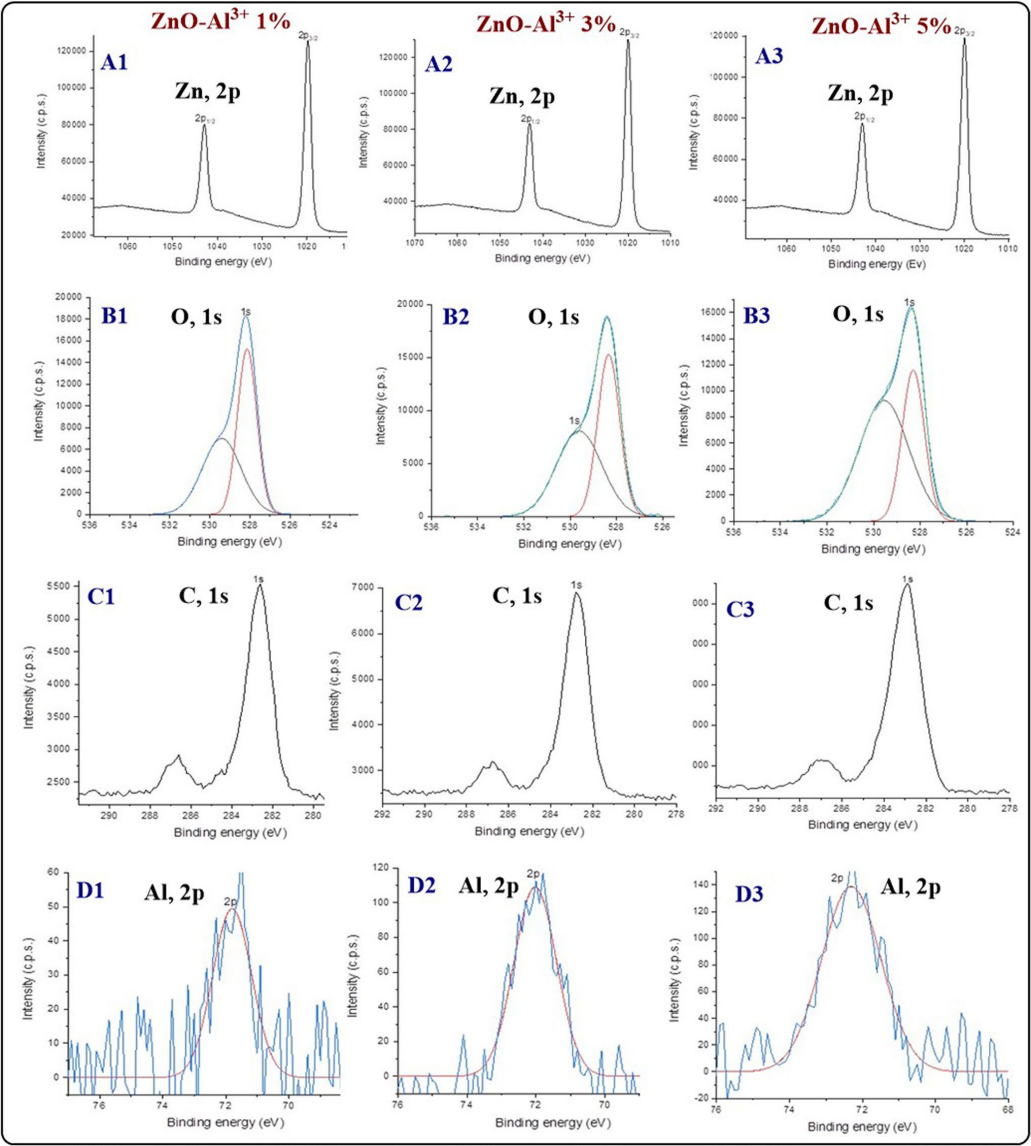
High resolution XPS spectra of AZO NMs at different doping levels (1, 3 and 5%). **A1**, **A2**, **A3** high resolution XPS spectra of Zn (1, 3 and 5%). **B1**, **B2**, **B3** high resolution XPS spectra of O (1, 3 and 5%). **C1**, **C1**, **C3**: high resolution XPS spectra of C (1, 3 and 5%). **D1**, **D2**, **D3**: high resolution XPS spectra of Al (1, 3 and 5%)

The results from XRD analysis show the crystalline structure, crystallite size, and doping effects on the crystalline structure of ZnO, and AZO, materials (Figs. 4, Figs. 5S, and 6S). Figure 4 shows the XRD diffractograms of AZO NMs. The NMs are crystalline with a wurtzite hexagonal structure. The XRD patterns of AZO NMs show a doping-dependent decreased intensity. In agreement with the HRTEM results, the incorporation of dopants into the ZnO lattice reduces the crystallite size and crystallinity of the materials because of the differences in the size of metal cations (Zn^2+^, Al^3+^) and defects in the ZnO lattice. The inset shows the shift of the main (101) peak to larger angles as the amount of Al^3+^doping increases. This shift to larger angles suggests the reduction in the interlayer spacing at ZnO along the (101) axis that results in the replacement of Zn^2+^(0.74 Å) with Al^3+^(0.53 Å) ions in the ZnO lattice [36].

#### Evaluation of the optical properties of AZO

We study the optical properties of AZO NMs using UV–Vis diffuse reflectance spectroscopy (Fig. 5). Our results indicate modification of the absorption properties of ZnO NMs as a function of doping level. The UV–Vis spectra of AZO NMs show a discrete absorption in the visible region (400–450 nm, Fig. 5A). This absorption increases proportionally to the amount of aluminum doping. Previous studies discuss the role of aluminum doping as a donor impurity in the ZnO lattice to expand its photo-response and improve its photocatalytic activity. The modification of the absorption properties of AZO NMS is due to the structural defects (lattice microstrain) and electronic modification due to the increase in the charge carrier concentration [37]. The increase in electron concentration leads to the filling of the lowest state in the conduction band (Burstein-Moss effect), increasing the Eg value of the doped ZnO NMs. We observe a slight increase in the Eg values of Al-doped NMs that is proportional to the increase in Al doping (Fig. 5B). Previous studies report an increase in the Eg values of aluminum-doped ZnO due to the generation of free carriers that accumulate in the conduction band, causing an increase in the optical band gap (Burstein-Moss effect) [19]. A different study reports the red shift in the absorption properties of AZO NMs due to the formation of new energy levels within the ZnO band gap [38].

#### X-ray photoelectron spectroscopy analysis (XPS)

**Fig. 7:**
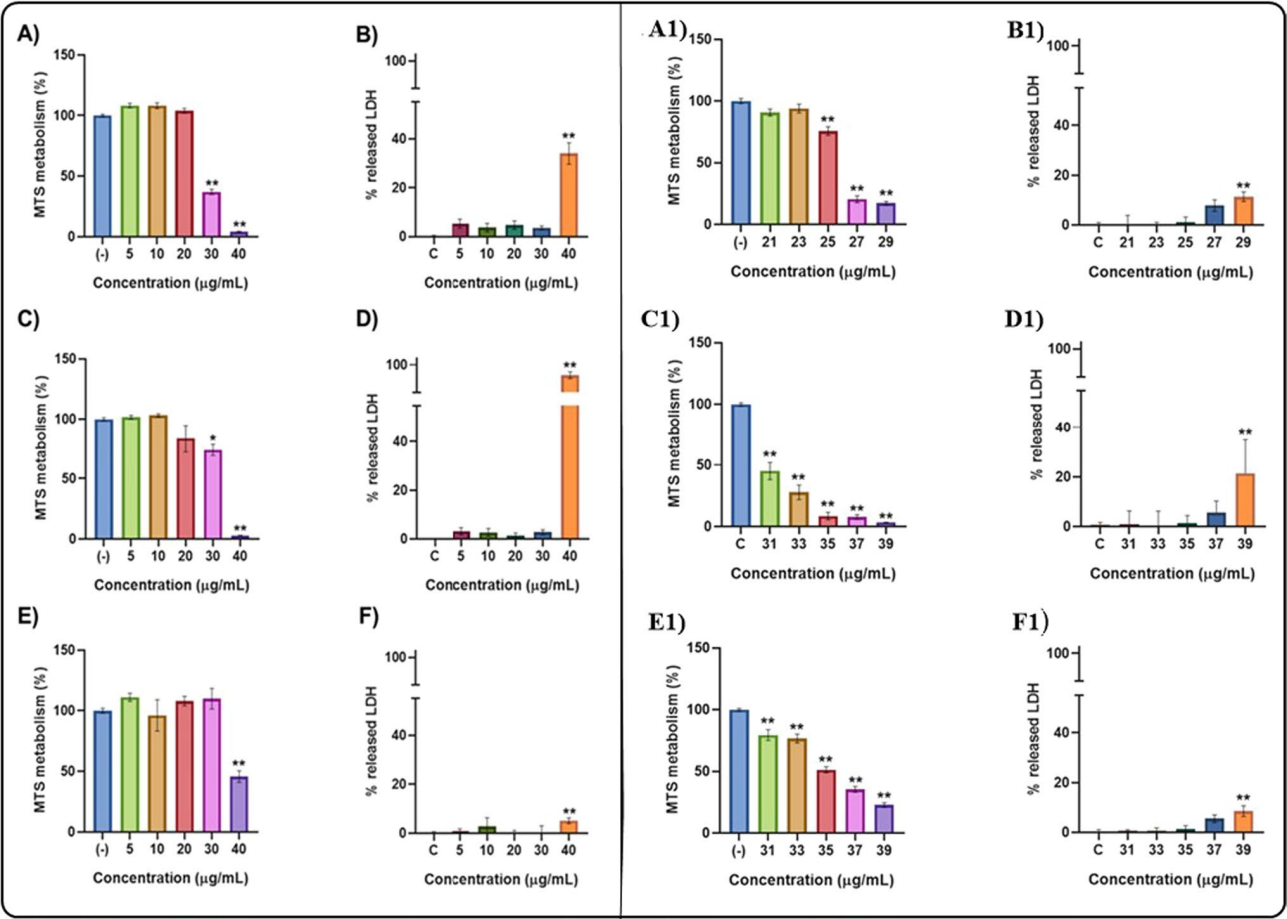
IC50 viability determination, using MTS and LDH assays, on SH-SY5Y cells after AZO NMs exposure to increasing concentrations (0 to 40 ug/mL) of ZnO NMs doped with Al with an atomic concentration of 1% (**A** and **B**), 3% (**C** and **D**), 5% (**E** and **F**). A refinement of cell viability determination after AZO NMs exposure is shown for Al doping concentrations of 1% (A1 and B1), 3%(C1 and D1), and 5%(E1 and F1). In all cases the LDH and the MTS assays were performed to determine the IC50 of NMs after 24 h of exposure; each bar represents the mean values of 3 different experiments ± SEM; *p < 0.05, **p < 0.01, ***p < 0.001 versus untreated cells. One way ANOVA*, *post hoc Bonferroni

XPS analysis resolves the chemical composition of ZnO, and AZO NMs. Figures 6 and 7S illustrate the general survey and high-resolution XPS spectra of the different NMs. The XPS survey spectra of ZnO (Fig. 7S A) denote the presence of Zn, O, and some impurities. In addition, high-resolution XPS spectra of ZnO show two peaks at 1043 and 1020 eV (Fig. 7S C) that correspond to Zn 2p1/2 and Zn 2 p3/2, respectively. 7S D exhibits two resonances (O 1 s) that correspond to, O-Zn (529 eV) and OH-Zn (530 eV) chemical species. There are also trace amounts of carbon species due to incomplete purification of NMs (287.6 eV) and adsorbed carbon from the atmosphere (283.6 eV; Fig. 7S E). Besides, the XPS analysis of AZO is very similar to ZnO except for the presence of the peak at 72.3 eV (Al 2p) that denotes the incorporation of aluminum into ZnO matrix (Fig. 6D). XPS analysis also shows a slight increase in the intensity of aluminum resonance as the amount of aluminum doping increases in the NMs.

Previous studies report the analysis of AZO NMs by XPS. The deconvoluted Al^3+^ peak consists of two peaks; one of them corresponds to the presence of Al^3+^ in the ZnO matrix (74.2 eV); the other is due to AlO(OH) species (76.8 eV) [38]. Yoo et al. studied the physicochemical and sensing properties of AZO NMs. XPS analysis confirms the insertion of Al into ZnO and the purity of the material with a single Al resonance at 74 eV that corresponds to the Al-O bond [36]. Our results show a single resonance for aluminum (~ 72 eV) characteristic of aluminum insertion into ZnO matrix.

#### Evaluation of the stability of suspended AZO NMs in DMEM (cell culture exposure medium)

The hydrodynamic diameter and zeta potential of the different AZO NMs were determined in water (Table S1) and DMEM medium with 1% FBS (Table 3) to investigate the suspension stability and behavior of the NMs during the biological interaction. As illustrated in Tables S1 and S2, the NMs under study show more stability in water than in DMEM medium. Table S1 shows the hydrodynamic diameter, the PDI and zeta potential of the different AZO NMs in water: 228.73 ± 18.57 nm, 0.471 and 30.37 ± 2.65 mV (1% doping); 218.77 ± 27.76 nm, 0.45 and 29.0 ± 2.32 mV (3% doping); 200.27 ± 19.81 nm, 0.41 and 31.03 ± 0.81 mV (5% doping). In water, AZO NMs present a slight decrease in the hydrodynamic size and PDI as the Al^3+^ doping concentration increases; meanwhile, the zeta potential values keep high and almost constant, denoting the stability of the materials in water.

**Table 3:** Physicochemical properties of the different NMs in DMEM medium

NMs	Effective density (g/cm^3^)		Hydrodynamic size (nm) / PDI		Zeta potential (mV)	
	T0	T1	T0	T1	T0	T1
ZnO–Al^3+^ 1%	1.05 ± 0.09	0.68 ± 0.06	36.16 ± 26.65 / 0.43	212.0 ± 13.71 / 0.50	− 10.82 ± 2.70	− 13.2 ± 0.85
ZnO–Al^3+^ 3%	1.29 ± 0.23	0.84 ± 0.15	18.32 ± 1.87 / 0.39	37.26 ± 24.9 / 0.52	− 7.58 ± 0.85	− 0.92 ± 1.17
ZnO–Al^3+^ 5%	1.08 ± 0.03	0.70 ± 0.02	196.24 ± 202.81 / 0.71	206.93 ± 32.90 / 0.60	− 10.27 ± 0.91	− 11.73 ± 0.61

Moreover, we estimate the stability of the different AZO NMs in the DMEM culture medium (Table 3). In addition, we calculate the effective density (ρ_EV_) of the NMs before (T0) and after (T1) their exposure to cells. The materials with the highest ρ_EV_ values have the lowest stability as a function of time in the exposure media, indicating that these materials sediment over time. The ρ_EV_ of our materials is lower than the one encountered for ZnO in a previous study [33], supporting the formation of NMs-medium aggregates and dissolution in the exposure medium. The high polydispersity index values exhibited by the NMs under study result from the dynamic nature of agglomerate size and density due to dissolution. There is a decrease in the effective density of the AZO NMs after their exposure to cells; these data support the dissolution of the NMs during exposition. Because the entrapped media have a lower density than the NMs, the effective density of the aggregates decreases. Because of the dynamic nature of agglomerate size (see uncertainty values) and effective density (which are likely overestimated) due to dissolution, we use these results to illustrate the changes of the AZO NMs during the exposure to cells [33].

The stability of AZO NMs in the exposure medium (DMEM) was estimated by measuring the hydrodynamic size, PDI, and zeta potential of the particles, immediately after preparing the suspensions (T0), and 24 h after preparation (T1). The values of hydrodynamic size, PDI, or zeta potential of AZO at T0 were: 36.16 ± 26.65 nm, 0.43, and − 10.82 ± 2.70 mV (1% doping); 18.32 ± 1.87 nm, 0.39 and − 7.58 ± 0.85 mV (3% doping); 196.24 ± 202.81 nm, 0.71 and − 10.27 ± 0.91 mV (5% doping). At T1 hydrodynamic size, PDI or zeta potential of AZO values were: 212.0 ± 13.71 nm, 0.50 and − 13.2 ± 0.85 mV (1% doping); 37.26 ± 24.9 nm, 0.52 and − 0.92 ± 1.17 mV (3% doping); 206.93 ± 32.90 nm, 0.60 and − 11.73 ± 0.61 (5% doping).

Our results indicate the formation of AZO (5%) aggregates. All the AZO-DMEM suspensions present a polydisperse population given by their PDI. In T1, the aggregation in the three different suspended NMs was observed compared with the T0. The zeta potential values remark on the low stability of the AZO NMs in the DMEM medium. In particular, AZO (3%) shows a significant stability loss within the exposure time. The hydrodynamic diameter and effective density contribute to the NMs sedimentation [32]. Besides, for a better understanding of the bioactivity of AZO NMs, it is necessary to consider protein corona formation during AZO NMs and DMEM interaction.

The protein corona composition confers biological identity to NMs and probably directs further interactions with proteins, cells, and tissues [38]. A dynamic exchange of corona components occurs resulting from the differences in abundance and diffusion rates of proteins [39]. Together, the protein corona interactions and the AZO NMs properties contribute to the differences in suspension behavior ( ρ_EV_ values, agglomeration size, PDI, and zeta potential) of these NMs over time.

We are aware that the surface functionalization of AZO NMs contributes to enhanced stability in suspension; however, the main applications of these materials seek enhanced catalytic activity in the solid state, decreasing activity with functionalization. Thus, in this study, we evaluate the behavior of naked materials (as fabricated in the solid state) to investigate their potential to harm living organisms.

### Cell viability and inhibitory concentration 50 calculation

We evaluated the potential cell toxicity using SH-SY5Y cells from the exposure to AZO (1, 3, and 5% at. % Al/Zn), measuring cell viability by MTS and LDH assays. Figure 7 shows the viability results after 24 h exposure to different concentrations of AZO NMs. As a result of AZO NMs exposure, SH-SY5Y cells showed a significant decrease in the MTS metabolism in the 30 and 40 μg/mL concentrations in the 1% and 3% doping (Fig. 7; A and C); meanwhile, the 5% doping concentration showed an effect only at 40 μg/mL (Fig. 7; E). For the LDH assay, all the doping concentrations showed a significant LDH release in the 40 μg/mL concentration (Fig. 7; B, D, F).

Given that the MTS assay showed a decrease in viability at lower concentrations than the LDH assay after the exposure to AZO NMs in cells, we estimated the IC50 values using the MTS assay results. Figure 7 show the results of the exposure of SH-SY5Y cells to different concentrations of AZO NMs evaluated by MTS (A1, C1, E1) and LDH assays (B1, D1, F1). Table 4 shows the calculated IC50 of each AZO NMs: 25.79 μg/mL (1% Al^3+^ doping); 31.06 μg/mL (3% Al^3+^ doping); 35.48 μg/mL (5% Al^3+^ doping). For AZO materials, IC50 increases as Al doping increases. The decreased toxicity of Al-doped materials can be related to their lower solubility, as previously reported by [17].

**Table 4:** Inhibitory concentration 50 and hemolytic activity of AZO NMs

Material	IC50 (µg/mL)	Hemolytic activity (%)*
ZnO	89.73	0.06
ZnO–Al^3+^ 1%	25.79	0.15
ZnO–Al^3+^ 3%	31.06	0.16
ZnO–Al^3+^ 5%	35.48	0.19

*Hemolytic activity of AZO NMs is expressed as the percentage mean, non -significant changes observed. Kruskal–Wallis, post hoc Dunn´s

**Fig. 8:**
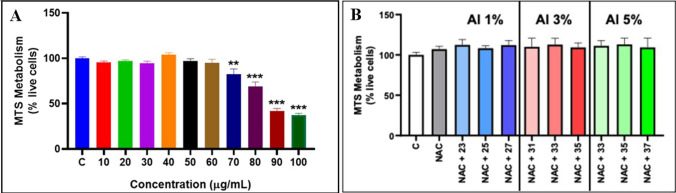
(**A**) Cell viability determination after ZnO NMs exposure. SH-SY5Y cells were exposed to different concentrations of ZnO NMs. The MTS assay was performed to determine the IC50 of NMs after 24 h of exposure. (**B**) Effect of the co-exposure between NAC and AZO NMs in SH-SY5Y cells. After the co- exposure to NAC and AZO NMs the viability of SH-SY5Y was determined by MTS assay. Each bar represents the mean values of 3 different experiments ± SEM; **p < 0.001, ***p < 0.0001 versus untreated cells. One-way ANOVA, post hoc Bonferroni

We also evaluated the cytotoxic effect of ZnO NPs on the SH-SY5Y cells to compare the biocompatibility of bare and doped ZnO NMs. Figure 8A illustrates the viability of SH-SY5Y cells as a function of exposure to different ZnO concentrations. The cytotoxic damage of bare ZnO occurs at concentrations higher than 70 µg/mL. We use these results to determine the IC50 value, which results in a concentration of 89.73 μg/mL for the ZnO NMs. This result indicates a higher biocompatibility of bare ZnO than doped ZnO NMs. It also points out the contribution of Al (doping element) in the cytotoxicity of the NMs.

We exposed the SH-SY5Y cells to increasing concentrations of aluminum ions (using aluminum (III) nitrate as an aluminum source) to determine the IC50 of this ion. Our results indicate that the IC50 of aluminum is 80.83 µg/mL. This value is higher than the encountered for AZO NMs and suggests the additive toxicity of Zn and Al in the AZO NMs. Furthermore, these results support the controlled release of ions (Zn^2+^ and Al^3+^) inside the cells. The controlled release contributes to increased activity, avoiding the precipitation of aluminum ions at physiological pHs. We previously stated that the acidic pH inside the cells results in the dissolution of AZO NMs and the release of the ions. Some other authors described the dependence on the chemical composition of aluminum precursor and its bioactivity. The lixiviation of aluminum ions and their bioactivity diminishes by the precipitation of this ion at physiological pH [40].

Considering the differences in the IC50 values observed for bare and doped ZnO NMs, we examined the possible contribution of oxidative damage as an adverse effect of exposure to AZO NMs. The co-exposure between N-acetyl cysteine (NAC) and the AZO NMs (1, 3, 5% of doping at. % Al/Zn) in all the concentrations showed the recovery of the cell viability approximately to 100% (Fig. 8B). These results corroborate that the cytotoxic damage of AZO NMs is related to the generation of oxidative damage in the SH-SY5Y cells.

The mechanism by which aluminum (Al) exerts toxicity to humans is not well understood. Al is not prone to change oxidation states; however, lately, this element is considered an oxidant that promotes the Fenton reaction, resulting in oxidative stress in human beings. A recent study points out that Al promotes the Fenton reaction by forming Al-superoxide radical complexes [30]. These complexes can increase oxidative stress in biological systems functioning as promoting agents of the Fenton reaction. Furthermore, Al is a strong Lewis acid with a high affinity to oxygen-containing functional groups (phosphates, carboxylic groups), forming complexes involved in the pro-oxidant activity of Al.

Our results show that SH-SY5Y cells can engulf NMs; inside the cell, these NMs tend to dissolve when exposed to the inner cell´s environment, thereby releasing metal ions responsible for oxidative damage. However, this controlled dissolution can be modulated by changes in their composition (doping level), shape, or surface modification, favoring their biocompatibility or biomedical application [41]. Recent studies remark on the importance of investigating the intracellular degradation of NMs to produce NMs with cancer-selective toxicity [42]. So far, iron-containing materials are the most used and studied. Nevertheless, ferric ions incorporate into the natural metabolism of the cells, reducing biomedical activity. In this regard, Al is an exogenous element to the human body, proven to generate oxidative stress in cells. As previously discussed, the synthesis route explored in this study allows for a facile modulation of the properties of AZO NMs. Thus, further studies aim to tune the properties of AZO NMs to explore their bioactivity (modulated dissolution and ROS generation) for controlled cancer cell toxicity.

Some other studies discuss the metal (Fe, Cu, Zn) mediated oxidative stress related to neurodegenerative disorders (Alzheimer's and Parkinson's diseases). Increasing iron (Fe) or copper (Cu) concentration induces free radical formation via the Fenton reaction [43]. The cell redox balance (equilibrium) depends on Fe and Cu redox couples. Furthermore, low zinc levels protect against amyloid-ꞵ toxicity, whereas increased zinc levels could trigger neuronal death and amyloid deposition. Our results are in agreement with oxidative damage of SH-SY5Y cells due to metal ion mediated oxidative stress. Furthermore, NAC is an effective antioxidant to prevent the oxidative damage induced by (Al^3+^, Zn^2+^) ions. Previous reports point out the antioxidant activity of NAC [13, 44]. NAC supports the synthesis of glutathione (GSH), an essential substrate to induce enzyme catalyzed oxidant scavenging and protein repair processes.

### Evaluation of the hemolytic activity of ZnO and AZO NMs

**Fig. 9:**
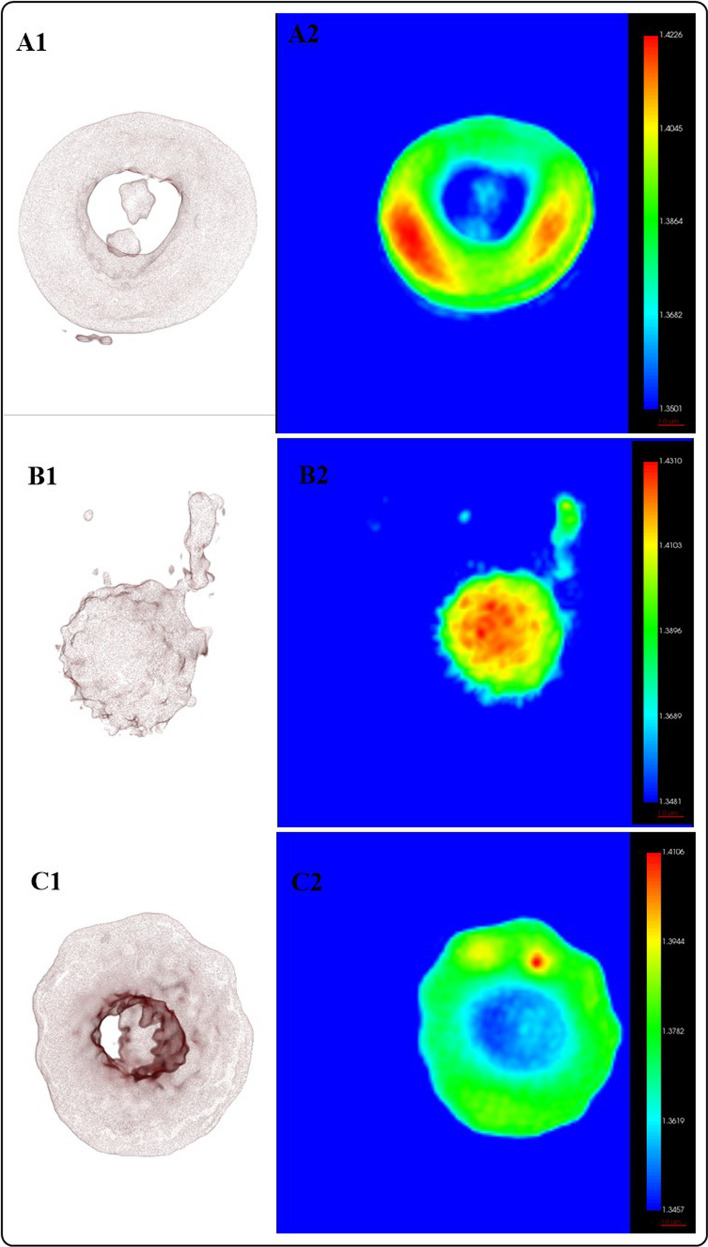
Evaluation of the interaction of AZO NMs with RBCs by HTM. **A** control group; **B** RBCs interacting with ZnO NMs; **c** RBCs interacting with AZO1% NMs. (1) 3D reconstruction of cells. (2) 2D optical phase projection images

**Fig. 10:**
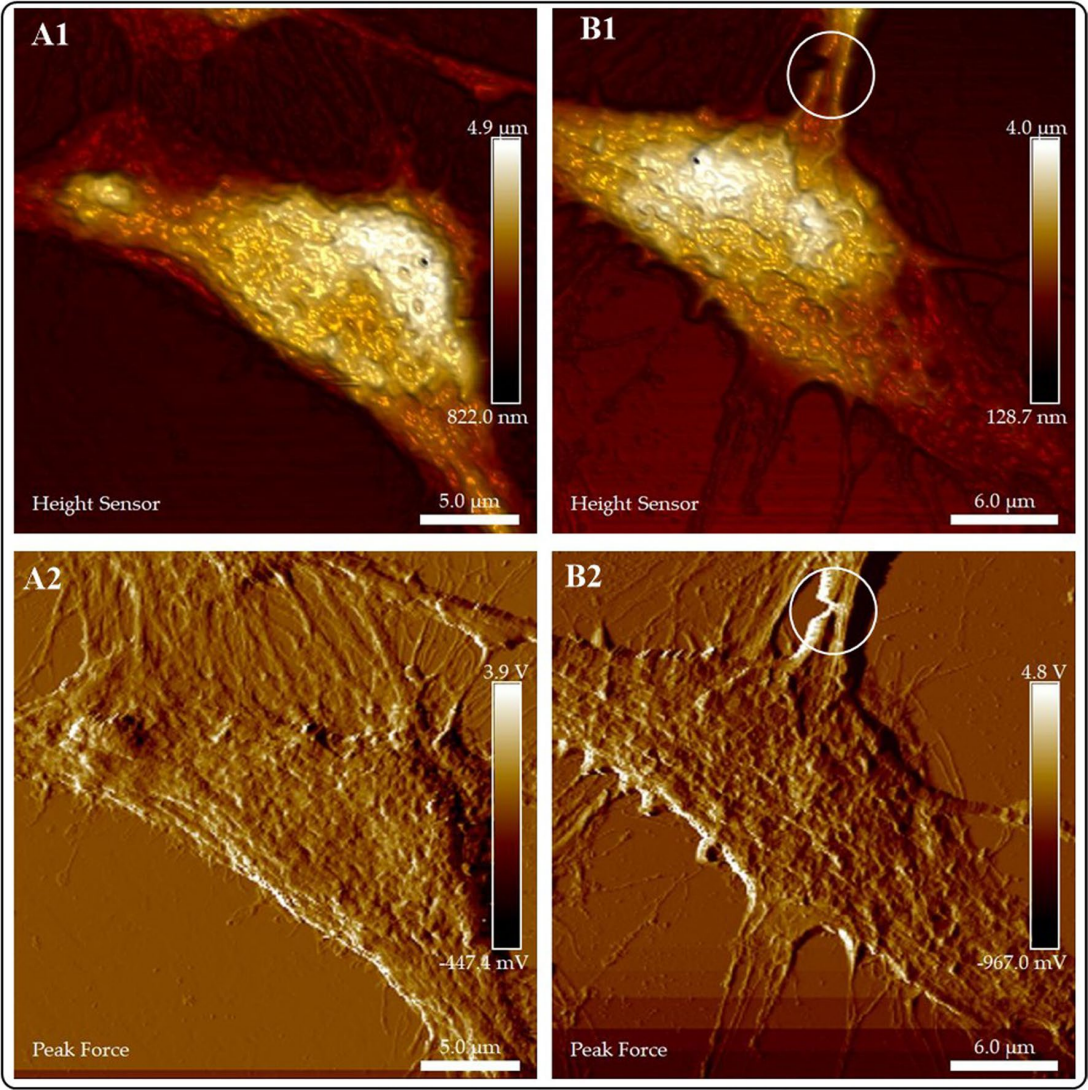
Evaluation of the interaction of AZO NMs with SH-SY5Y cells by AFM. **A** control group; **B** SH-SY5Y cells interacting with AZO (ZnO–Al^3+^. 1% 10 mg/mL), the white circle denotes neurite connection disruption

In contrast with the cytotoxic activity of ZnO and AZO NMs exerted in neuroblatoma (SH-SY5Y) cells, our results (Table 5, Fig. 9 and 10S) indicate the no hemolytic activity of the NMs under study. The hemolytic activity is lower than 0.2% suggesting the biocompatibility of the materials with healthy red blood cells.

**Table 5:** Quantitative characterization of RBCs based on analysis of the RI from HTM

Parameter	Control	ZnO treated	AZO treated		
			1%	3%	5%
Volume (µm^3^)	101.7391	97.3715	160.5849***	138.4099***	125.8210***
Surface area (µm^2^)	170.2822	127.1309***	197.8379**	191.1168	175.4617
Mean RI	1.3771	1,3829	1.3654***	1.3670***	1.3678***
Hemoglobin concentration (pg/µm^3^)	0.2894	0.3283	0.2112***	0.2223***	0.2165***
Dry mass (pg)	28.8001	31.1020	33.4691***	30.3246	26.7778
Sphericity	0.6261	0.8022***	0.7251***	0.6834*	0.6995***

Each value represents the mean values of 3 different experiments; *p < 0.05, **p < 0.01, ***p < 0.001 versus control. Kruskal–Wallis post hoc Dunn´s

To further investigate the interaction of RBCs with the NMs under study, we analyze the morphology (shape, size) and composition (hemoglobin content, dry mass) of the RBCs (treated and no-treated). Our results indicate no significant changes in the morphology or composition of the RBCs exposed to the NMs under study (Table 5 and Fig. 9). The physical (volume, surface area, and sphericity) characteristics of the RBCs in the control group correspond to the properties of healthy cells following previously published results [45, 46]. For the treated groups, we observed morphological changes (the presence of discocytes, echinocytes, and spherocytes) but not chemical alterations (Hb content, dry mass). These results indicate that the changes in the morphology of the treated RBCs might be due to changes in the media osmotic pressure due to ion lixiviation of the NMs. Since the dose IC50 of ZnO is higher than AZO NMs, RBCs were exposed to higher concentrations of ZnO, resulting in more evident morphological changes. Regarding the exposure to AZO NMs, our results show more morphological changes with the group exposed to AZO 1% in accordance with the IC50 encountered for SH-SY5Ycells.

#### AFM evaluation of the interaction of SH-SY5Y cells with AZO NMs

**Fig. 11:**
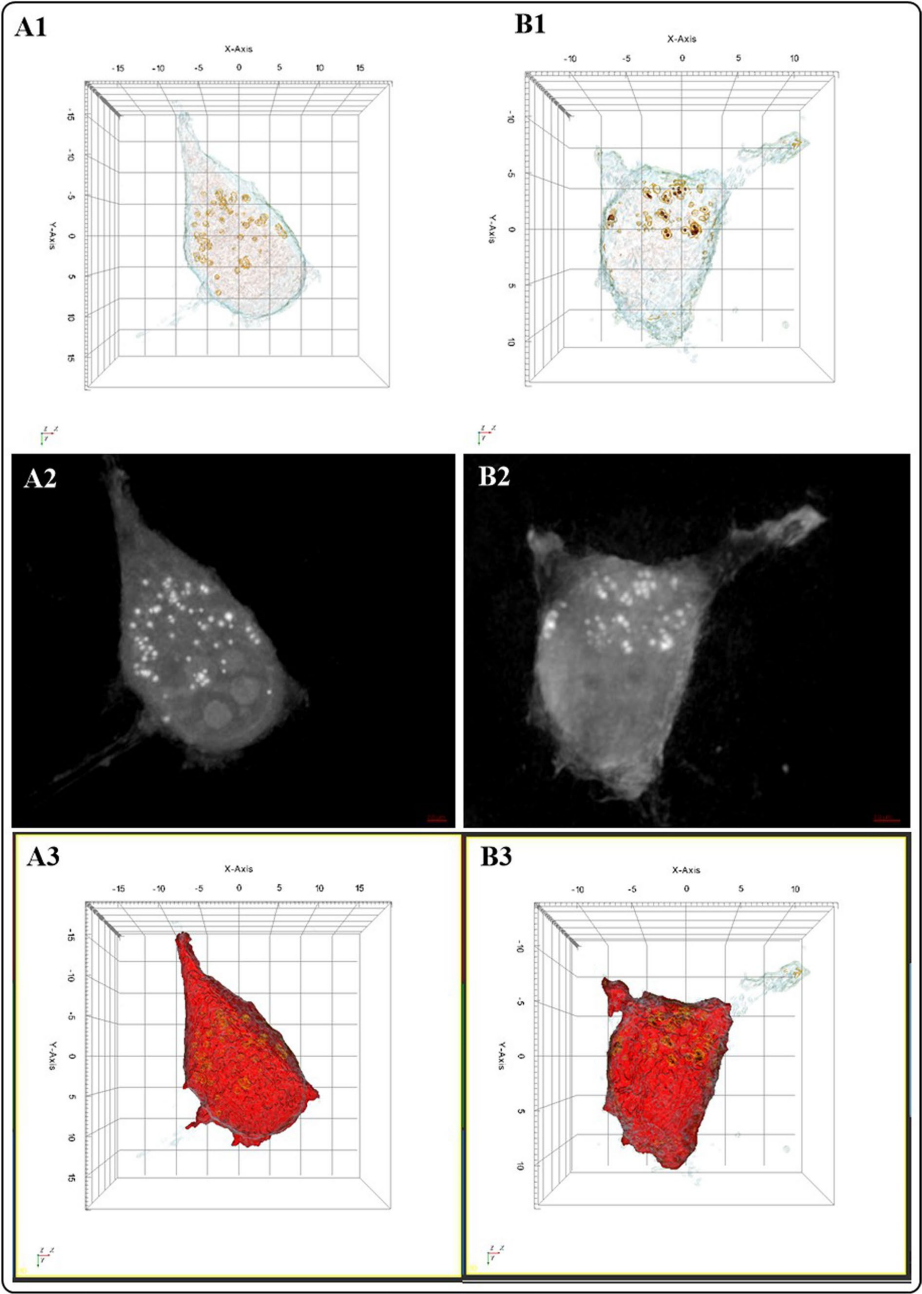
Evaluation of the interaction of AZO NMs with SH-SY5Y cells by HTM. **A** control group; **B** SH-SY5Y cells interacting with AZO NMs (ZnO–Al^3+^. 1% 5 mg/mL). (1) 3D reconstruction of cells. (2) 2D optical phase projection images. (3) Quantitative phase imaging quantification of lipid droplets

**Fig. 12:**
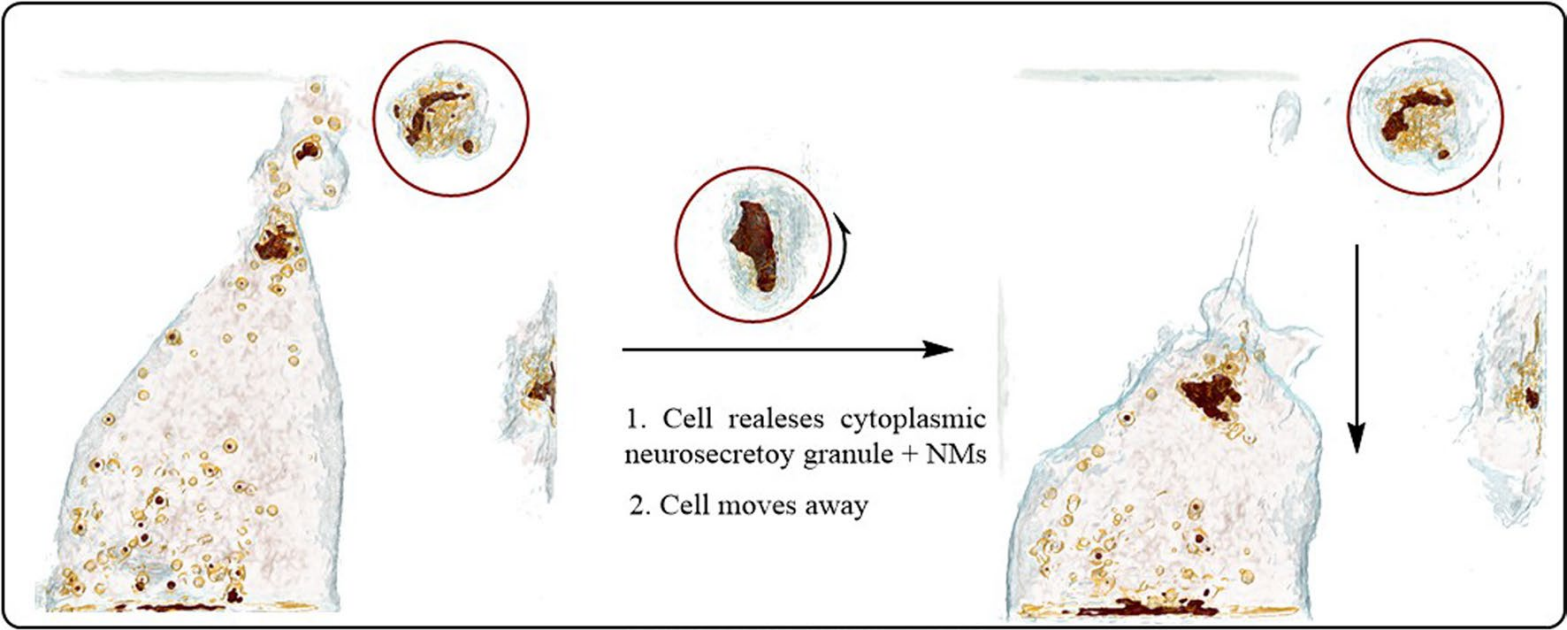
HTM imaging of living SH-SY5Y cells interacting with AZO NMs. Left side, the cell secretes NMs containing vesicle. Right side, cell moves away from secreted vesicle

For a better understanding of the mean of interaction between SH-SY5Y cells and NMs, we investigate the morphological changes of cells using optical (Fig. 11S) and AFM after NMs exposure**. **Figures 10, 11S–15S illustrate our results. The SH-SY5Y cells from the control group show their characteristic spindle morphology and multiple connections with nearby cells (Figs. 10A1, A2, and 11S). The measurements of cell height and width correspond to the size of SH-SY5Y cells previously analyzed by AFM (height, 5.23 µm; width, 8.35 µm) [47]. Images of SH-SY5Y control group cells also show the presence of cytoplasmic neurosecretory granules.

On the other hand, SH-SY5Y cells exposed to AZO NMs also exhibit spindle body morphology, but the neurite connection is damaged (white circle). AFM analysis also permits visualization of AZO NMs on the surface of cells (Figs. 12S A1-A4 and A1a to A4a). The presence of NMs in the cell´s surface limits the connection with surrounding cells (Figs. 14S B1, B2, B1b, B2b). Our results also show that the SH-SY5Y cells exposed to AZO NMs exhibit different morphologies (Spindle and stellar; Fig. 13S). The AZO-treated group shows an increase in the appearance of cytoplasmic neurosecretory granules (indicated with arrows).

#### HTM evaluation of the interaction of SH-SY5Y cells with AZO NMs

To complement the AFM study on the morphological changes of cells at high resolution, we evaluate morphofunctional changes in cells using HTM. While; AFM provides a detailed ultrastructural analysis, an inspection of the inner’s cell morphology and changes upon exposure to NMs is necessary to investigate the entry and final destination of NMs in the cells. Figures 11, 16S–18S illustrate the HTM analysis of SH-SY5Y cells and SH-SY5Y cells exposed to different doses of the AZO NMs.

Figure A1 (3D refraction index –RI- colored image, according to RI values) shows the spindle morphology of the SH-SY5Y cells. The 2D optical phase projection image of control and treated cells reveals the differences in cell components (Figs. 11 B1, B2, and B3) after exposure to NMs. The bright white dots correspond to lipid droplets, a cell component suitable as a biomarker of cell stress against exposure to exogenous agents. Previous studies demonstrate that an increase in lipid droplet biogenesis in non-adipose tissue is associated with oxidative stress and an increase in reactive oxygen species (ROS) due to exposure to pro-oxidant chemicals [48, 49]. Figure 11 A3 shows quantitative phase imaging quantification of lipid droplets. The 3D reconstruction of AZO-treated cells (Fig. 11 B1) shows slight changes in their morphology and the presence of NMs (red dots) inside the cells. Figure 11 B2 exhibits differences in lipid drop distribution and concentration. Our results show that NMs enter SH-SY5Y cells, causing an increase in lipid droplets; these droplets participate in the clearance of NMs from the cell

To perform quantitative imaging and analysis of SH-SY5Y cells, we use commercial software (Tomostudio, Tomocube Inc., Republic of Korea), as shown in Fig. 11 (A3, B3, C3). Table 6 shows the quantitative characterizations of the control group and AZO NMs treated groups. Following AFM analysis, we observe major morphological damage in cells exposed to AZO NMs; the cell volume of these cells decreases in comparison to the control group. Chronic exposure to aluminum has been linked to neurodegenerative disorders [50]. We can also observe an increment in the concentration of lipids and a decrement in dry mass content in treated groups. The endocytic nature of SH-SY5Y cells allows NMs access to the cell. Inside the cells, NMs exert oxidative stress damage; one of the biological responses of the cells to oxidative damage is to increase lipid production to transport the toxicant outside the cell [43].

**Table 6:** Quantitative characterization of SH-SY5Y cells based on analysis of the RI from HTM

Parameter	Control	AZO treated
Volume (µm^3^)	2225.9420	1180.1054
Surface area (µm^2^)	1208.6126	729.0367
Mean RI	1.3648	1.3664
Lipid concentration (pg/µm^3^)	0.2334	0.2564
Dry mass (pg)	519.6124	300.4768

A remarkable advantage of HTM is the analysis of living cells that allows visualizing the cell mechanisms of interaction with exogenous agents. Figure 12 and Fig. 18S A1 shows SH-SY5Y cell-secreting granules that engulf NMs. The NMs colored in red in the 3D reconstructed image. After secreting the NMs containing granules, the cell moves away (Fig. 12 and Fig. 18S A2). The 2D optical phase projection images (Figs. 18S B1 and B2) and bright-field images support the observations of the internalization of NMs and the clearance mechanisms that the cell use to avoid damage. There are not many studies regarding the metallo-neurobiology of oxidative metals (Fe) and non-oxidative metals (Zn, Al) in neurodegenerative disorders. This study shows the activity of neuronal cells clearing NMs that can be associated to pathological deposits in neurogenerative diseases.

Previous studies demonstrate the suitability of HTM imaging as a powerful strategy for biomedical research. For instance, Kang studied the interaction between NPs (Au, graphene) and immune cells. HTM demonstrates the internalization of NMs and increased lipid cell concentration [34]. A different study discusses that the treatment of BEAS-2B cells with MoS_2_ or WS_2_ NPs induces changes in the morphology and organelles of cells, which can be monitored by HTM [51]. A recent review article remarks that HTM is a robust strategy that provides objective measurements of morphology changes and dynamics in biomedicine [52].Last, HTM is suitable for performing quantitative imaging to evaluate alterations in cell´s contents. For example, Park et al., investigated the "in vitro" neurotoxic effects in SH-SY5Y cells of 1-methyl-4-phenylpyridinium ion (MPP +), a metabolite of 1-methyl-4-phenyl-1,2,3,6-tetrahydropyridine (MPTP), a chemical associated to Parkinson's disease development. HTM analysis showed morphological and biophysical alterations associated to apoptosis in the cells under study [53].

It is well known that the lack of standard procedures or standard control reagents for nanotoxicity assays result in a loss of consistency between experimental procedures. HTM is a flexible and powerful technique that allows to monitor the interaction of NMs and living cells in real time. Our results show that HT microscopy is a versatile technique suitable for monitoring the uptake, transformation, and clearance of NMs by cells (SH-SY5Y). Further studies will evaluate its suitability to monitor the changes induced in cells due to controlled metal (Zn^2+^, Al^3+^) ion lixiviation. Our results might be relevant for the development of nano-therapies for controlled cancer cell cytotoxicity and a better understanding of the biological response of neuronal cells to metal ion oxidative stress.

Numerous studies demonstrate the capacity of nanoparticles (NPs) to overcome the blood–brain barrier (BBB) [54]. Nowadays, many research efforts aim to apply this capacity of NPs to treat Central Nervous System (CNS) diseases (neurodegenerative diseases, brain cancer) [55]. ZnO NPs exhibit the capacity to translocate BBB. Kao et al. (2012) demonstrated the translocation of ZnO NPs to the brain after inhalation through the olfactory bulb [56]. Later, [57] reported that ZnO Nps induces neuroinflammation via the taste nerve translocation pathway. A recent study demonstrated that ZnO NPs relieve the brain damage caused by aluminum (III) chloride in an experimental Alzheimer's disease model [58]. Liu et al. used 3D brain organoids to evaluate the mechanism of ZnO cytotoxicity. They found that high concentrations of ZnO NPs are cytotoxic and that this cytotoxicity occurs by defective autophagy and excessive accumulation of intracellular Zn ions [59]. These examples remark on the promising advances for the treatment of CNS diseases using NP-mediated brain drug delivery. However, there is a need to optimize the design of NPs to avoid healthy cell cytotoxicity.

In this study, we use SH-SY5Y cells to evaluate the bioactivity of AZO NMs. We are aware of the limitations of using a 2D cell model. These systems do not represent the complex nature of human organs (or tumors) nor consider the surrounding microenvironment, the cell–cell, or the extracellular-cell matrix (ECM) interactions. However, there are advantages to 2D cultures, particularly their suitability to be examined by different microscopy techniques, allowing the 4D visualization of the interaction of exogenous agents and cells. On the other hand, nowadays, 3D cell models offer numerous advantages to advance nanomedicine or nanotoxicology discoveries [60, 61]. We are currently working on developing 3D systems (spheroids or organoids) to conduct further studies.

## Conclusions

This study shows the advantages of MW-assisted fabrication of ZnO and AZO NMs. Changes in the biocompatibility of the NMs depend on their composition and physicochemical properties. In this study, we combine classical toxicology assays (viability and oxidative stress modulation) and advanced microscopy to interpret the potential toxicity mechanisms by which SH-SY5Y cells interact with AZO NMs. AFM and HTM as complementary methods study the interaction of NMs and living cells. The high-resolution AFM images denote morphological changes and loss of connectivity in SH-SY5Y cells exposed to AZO NMs. In addition, HTM imaging shows the internalization of AZO NMs in SH-SY5Y cells. We demonstrate that HTM is a powerful tool for investigating the uptake, transformation, interaction, and destination of NMs in SH-SY5Y cells. It is also a straight forward strategy to study biological processes in living cells and in *real time*.

## Limitations and further perspectives

In this study, we investigate the modification of the physicochemical properties of AZO NMs as a function of the Al^3+^ doping level. To our knowledge, no previous studies exist regarding the microwave-activated solvothermal synthesis of AZO NMs. In this study, we use aluminum nitrate as the aluminum precursor, but further studies will focus on a different (aluminum acetate, aluminum acetylacetonate) precursor to investigate the possibility of increasing the solubility limit of Al^3+^ in the ZnO lattice. Our results indicate the formation of AZO materials with slight differences in the aluminum content that do not follow the experimental setup, probably due to the limited solubility of aluminum nitrate in benzyl alcohol. Furthermore, metal precursors (such as acetates or acetylacetonate) react with benzyl alcohol, forming binary or ternary metal oxides (Al_x_Zn_1-x_O) in a molecular controlled manner. In addition, we want to systematically study the nano interactions of diverse AZO NMs with neuroblastoma cells in real time and reveal more details on the neurotoxicity mechanisms of aluminum or aluminum-containing compounds. Also, the molecular mechanism of the AZO NM's cytotoxic damage needs to be explored (ROS induction by misfolded proteins, inhibition of the NFE2-related factor 2, disruption of the mitochondrial electron transport). Besides, the molecular pathways associated with the neurite connection damage observed for the AZO-treated groups of SHSY-5Y cells (AFM images) need a systematic investigation. One of our aims is to develop AZO NMs with specific cytotoxicity for neuroblastoma cells.

### Supplementary Information

Additional file 1 (DOCX 7133 KB)

## Data Availability

The authors declare that the data supporting the findings of this study are available within the paper and its Supplementary Information files. Should any raw data files be needed in another format they are available from the corresponding author upon reasonable request.
