# Supplementary material for: Aluminum enhances the oxidative damage of ZnO NMs in the human neuroblastoma SH-SY5Y cell line
Source: Discov Nano. 2024 Feb 26;19(1):36. doi: 10.1186/s11671-024-03973-2 (PMC10897122; doi:10.1186/s11671-024-03973-2)
Supplement: Supplementary file 1 — Additional file 1 (DOCX 7133 KB) [file 11671_2024_3973_MOESM1_ESM.docx]

**Supplementary Information**

**Aluminum enhances the oxidative damage of ZnO NMs in the human neuroblastoma SH-SY5Y cell line.**

Arturo Jimenez-Chavez^1^‡, Gladis Pedroza-Herrera^2^‡, Israel Betancourt-Reyes^3^, Andrea De Vizcaya Ruiz^1,6^, David Masuoka-Ito^4^, Juan Antonio Zapien^5٭^, Iliana E. Medina-Ramirez^2٭^

^1^ Departamento de Toxicología, Centro de Invetigación y Estudios Avanzados de IPN (CINVESTAV-IPN), Ciudad de México, México.

^2^ Department of Chemistry, Universidad Autónoma de Aguascalientes. Av. Universidad 940, Aguascalientes, Ags, Mexico

^3^ Instituto de Investigaciones en Materiales, Universidad Nacional Autonoma de México, México.

^4^ Department of Stomatology, Universidad Autónoma de Aguascalientes. Av. Universidad 940, Aguascalientes, Ags., Mexico

^5^ Department of Materials Science and Engineering, City University of Hong Kong, Hong Kong SAR, PR China

^6^Department of Environmental and Occupational Health, Program in Public Health, University of California Irvine, Irvine, CA, USA.

Correspondence: [iemedina@correo.uaa.mx](mailto:iemedina@correo.uaa.mx); [apjazs@cityu.edu.hk](mailto:apjazs@cityu.edu.hk)

‡ These authors contributed equally


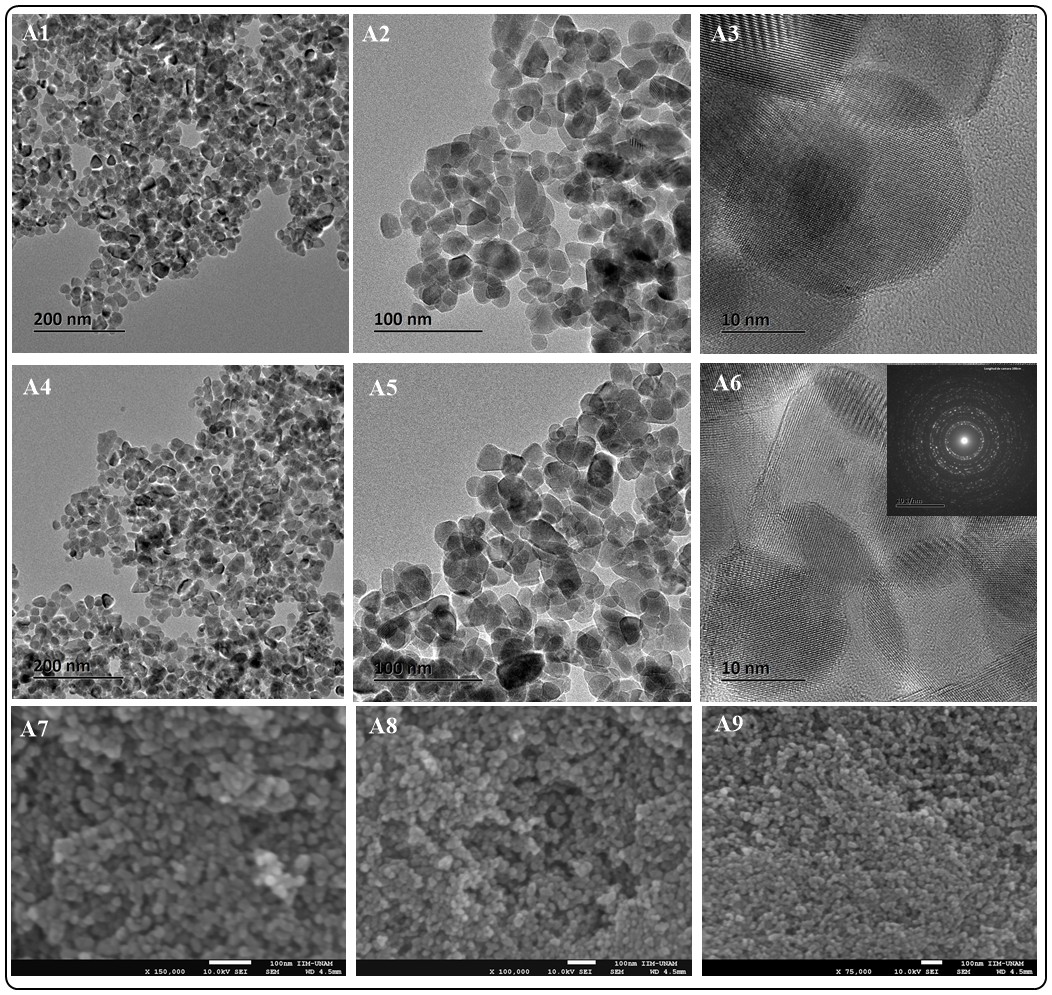


**Figure 1S**. TEM, HRTEM and SEM analysis of bare ZnO NMs. A1, A2, A4, A5) TEM analysis of ZnO illustrating the uniform size and morphology of the NMs. A3, A6) HRTEM analysis of ZnO NMs illustrating the small size of the NMs, the inset (A6) SAED pattern. A7-A9) SEM analysis of ZnO NMs show the spherical morphology of the NMs and uniform size.


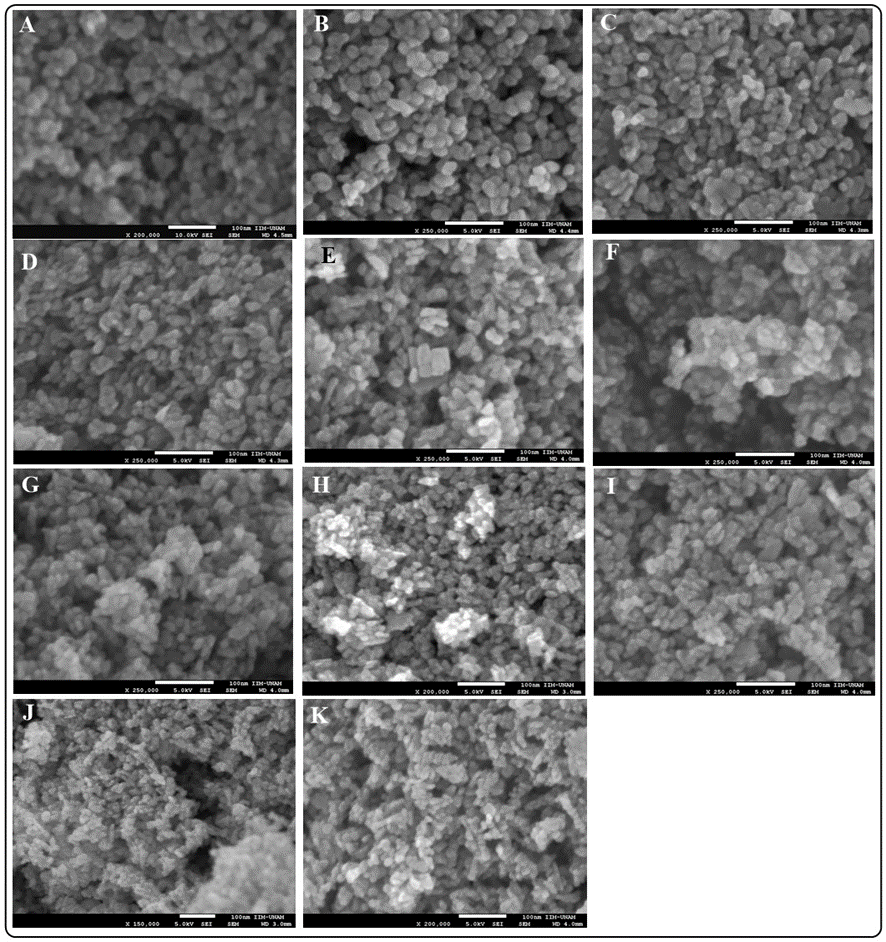


**Figure 2S.** Low-resolution SEM micrographs of ZnO (A) and AZO (0.5, 1.0, 1,5, 2.0, 2.5, 3.0, 3.5, 4.0, 4.5 and 5 at. % Al/Zn) B –K.


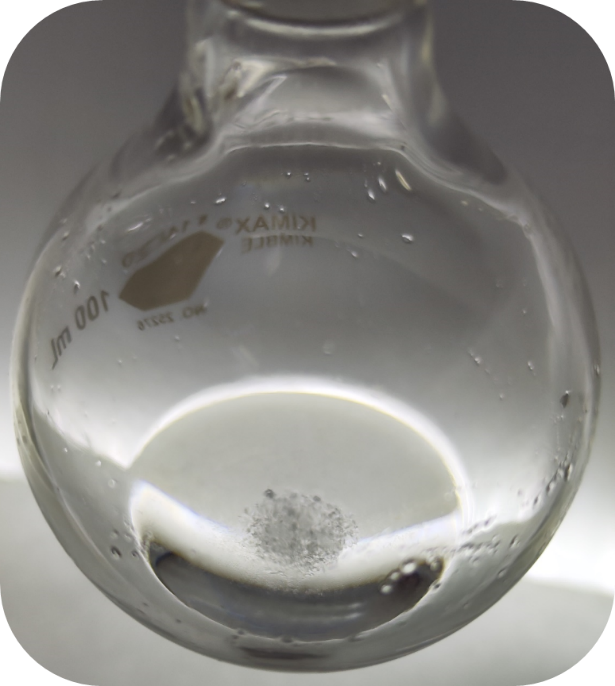


**Figure 3S.** Schematic representation of aluminum (III) nitrate precursor in benzyl alcohol before reaction. Aluminum nitrate precursor is not highly soluble at room temperature in the reaction solvent.


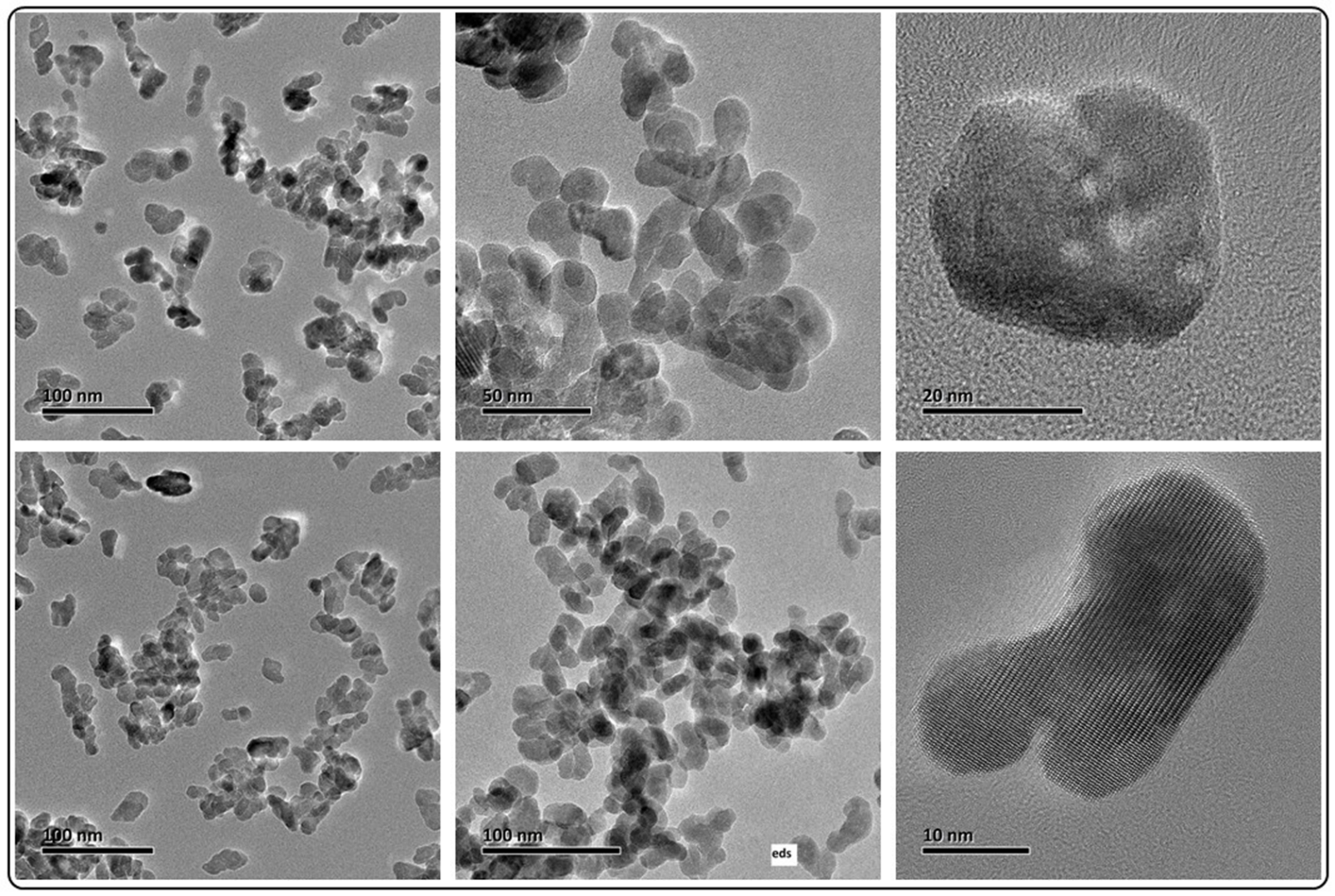


A6

A5

A4

A3

A2

A1


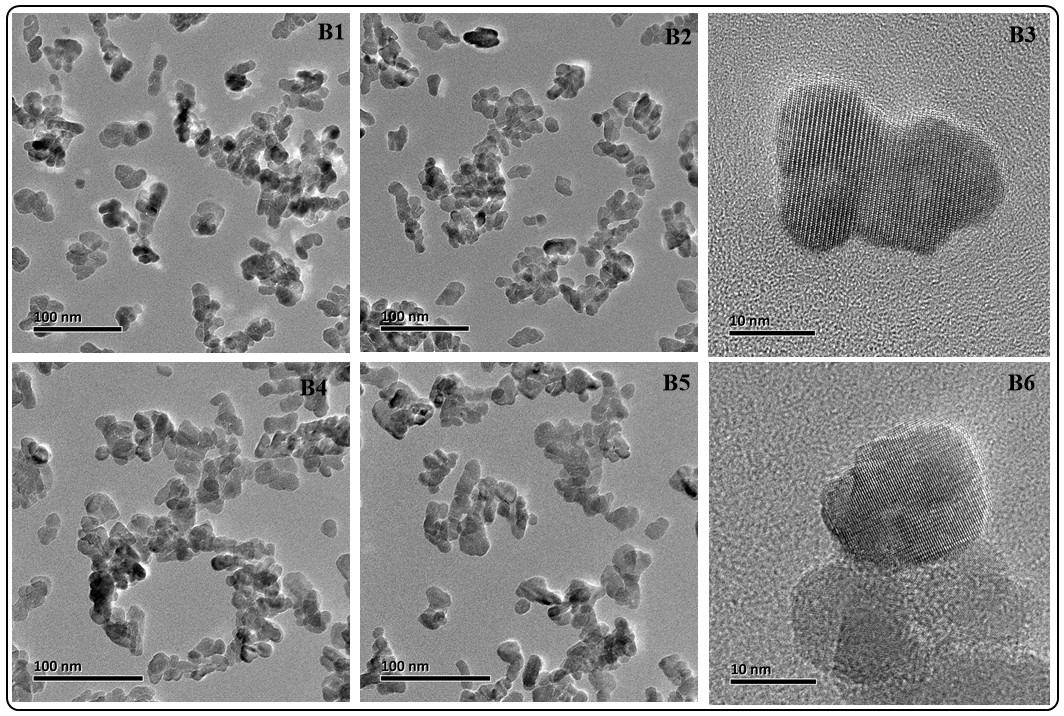


B6

B5

B4

B3

B1!1

B2

100 nm


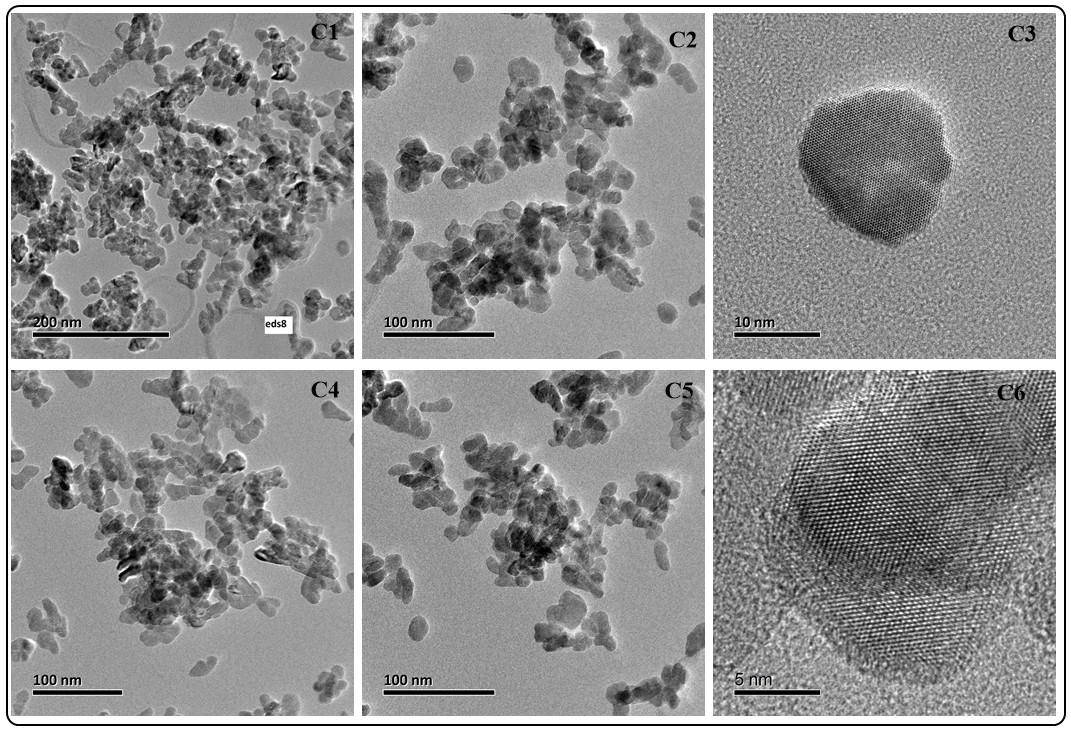


C6

C5

C4

C3

C2

C1

**Figure 4S.** TEM analysis of AZO NMs for different Al doping levels of 1, 3, and 5 at. %. A1 to A6: 1 at. % Al; B1 to B6: 3 at. % Al; and C1 to C6: 5 at. % Al.

**Figure 5S.** XRD analysis of AZO NMs. Black, AZO 0.5% (at% Al/Zn), Red, AZO 5% (at% Al/Zn). By comparing the spectrum of the NMs, it is evident the incorporation of the dopant into the ZnO matrix, rendering a decrease in the crystal size (lower intensity and wider peaks) and shifting of the peaks

**Figure 6S**. XRD analysis AZO NMs. From A to J. 0.5, 1.0, 1.5, 2.0, 2.5, 3.0, 3.5, 4.0, 4.5 and 5.0 ZnO-Al^3+^at. % (Al/Zn).


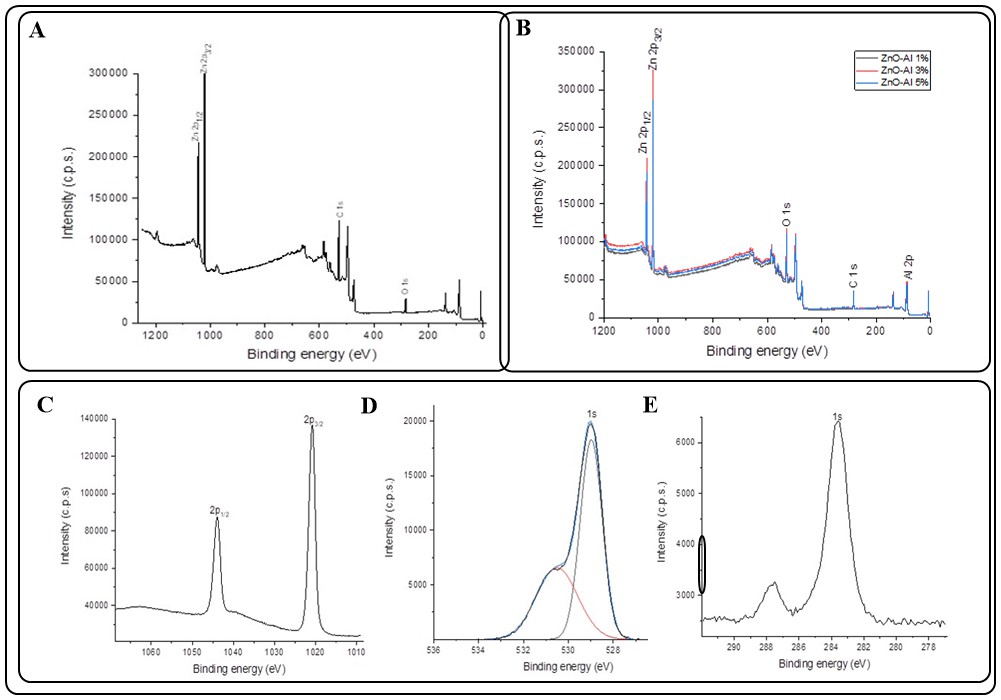


**Figure 7S.** XPS analysis of bare and doped ZnO NMs. A) Survey XPS spectra for ZnO NMs. The NMs are composed of Zn, O and trace amounts of carbon impurities. B) Survey XPS spectra of Al:ZnO. The spectra show that the main components of the NMs are Zn and O; however, aluminum dopant is also present, demonstrating its incorporation into ZnO NMs. C-E) High Resolution-XPS spectra of ZnO: Zinc (C), Oxygen (D), and Carbon (E).

**
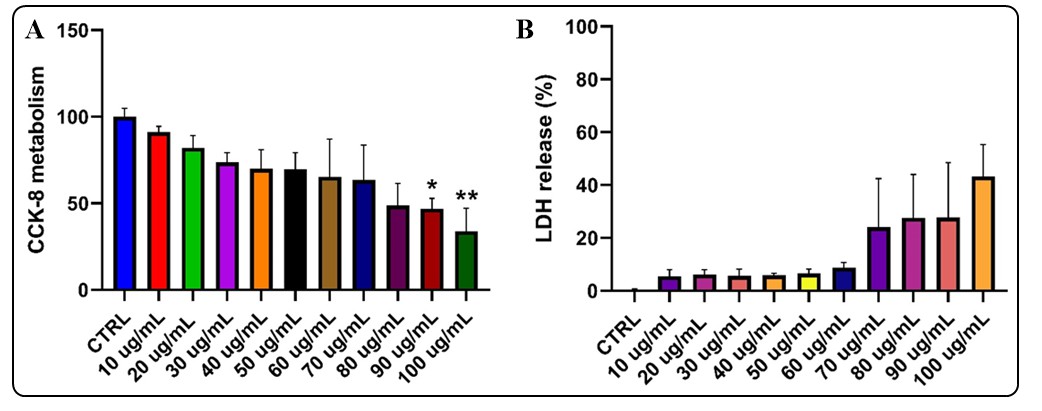
**

**Figure 8S.** Cell viability determination for SH-SY5Y cells after Al ions exposure. Results were obtained after 24 h of exposure to different concentrations of Al. Data from 1 experiment by triplicate. Mean +/- SEM. *p=0.05, **p=0.01 vs control. One way ANOVA, *pos hoc* Bonferroni. A) CCH-8 assay. B) LDH release assay.

**
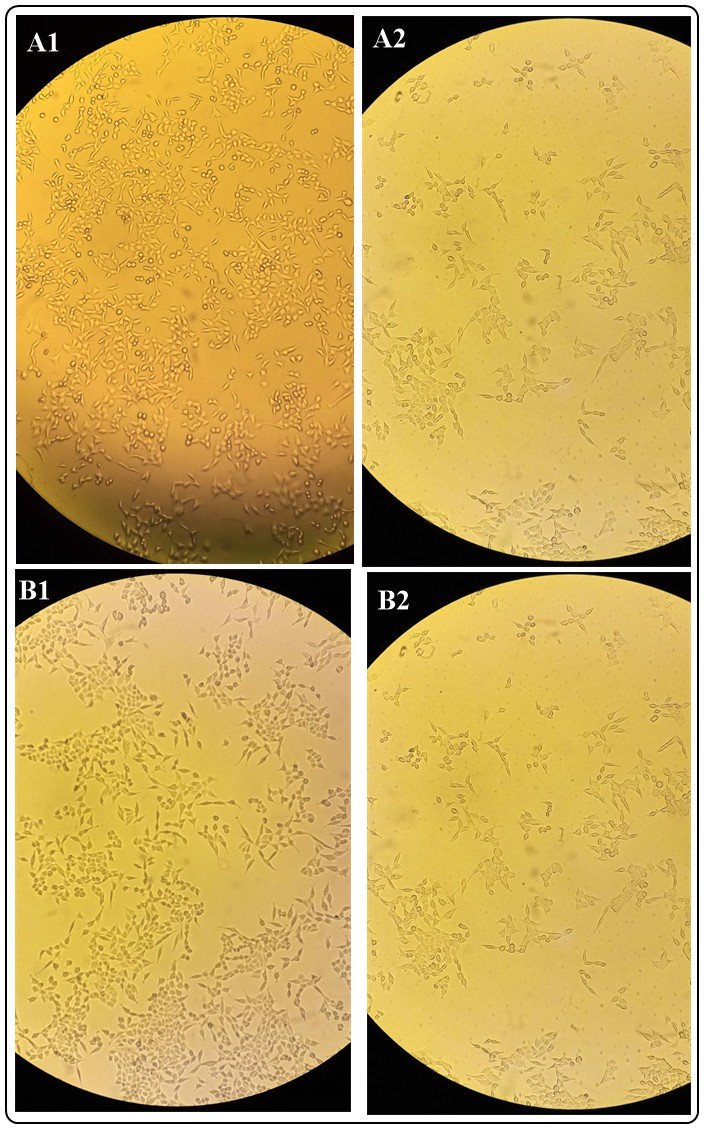
**

**Figure 9S.** Optical microscopy analysis of SH-SY5Y cells. A) Control group showing normal morphology f

or this cell line. B) Aluminum treated group showing normal and morphological changes**.**

**
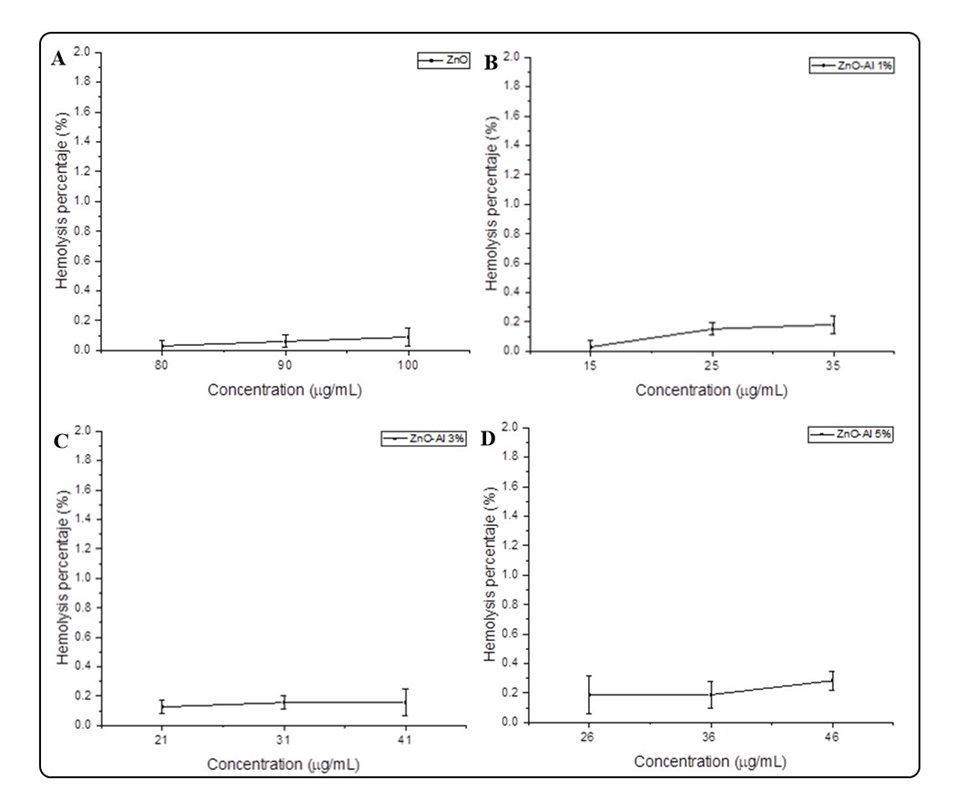
**

**Figure 10S.** Hemolytic activity of ZnO and AZO NMs.

**
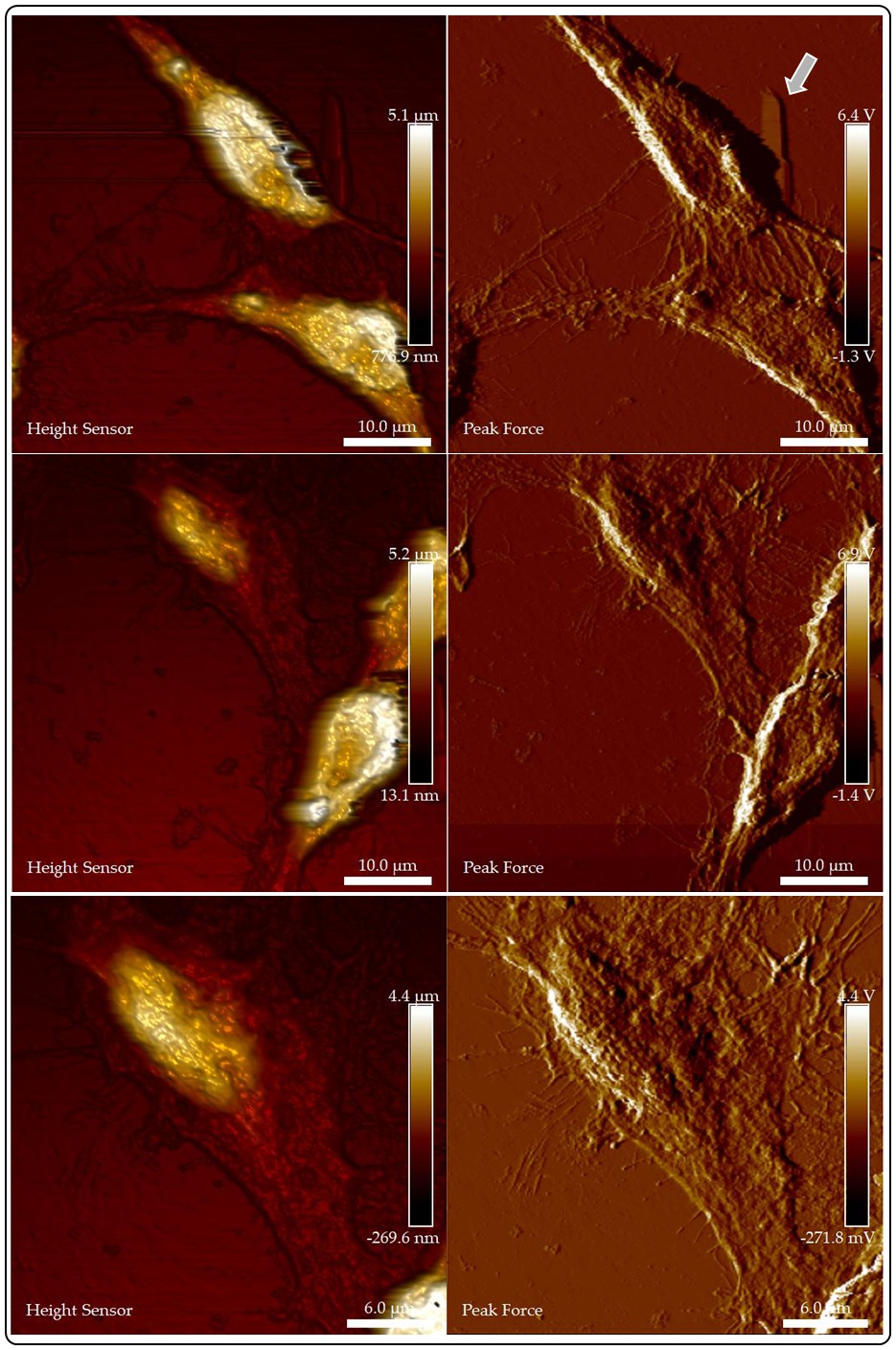
**

**Figure 11S.** AFM Analysis of the interaction of NMs and SH-SY5Y cells. AFM images of SH-SY5Y cells, control group. AFM images showing cells with normal morphology. The white arrow indicates the presence of cytoplasmic neurosecretory granules.

**
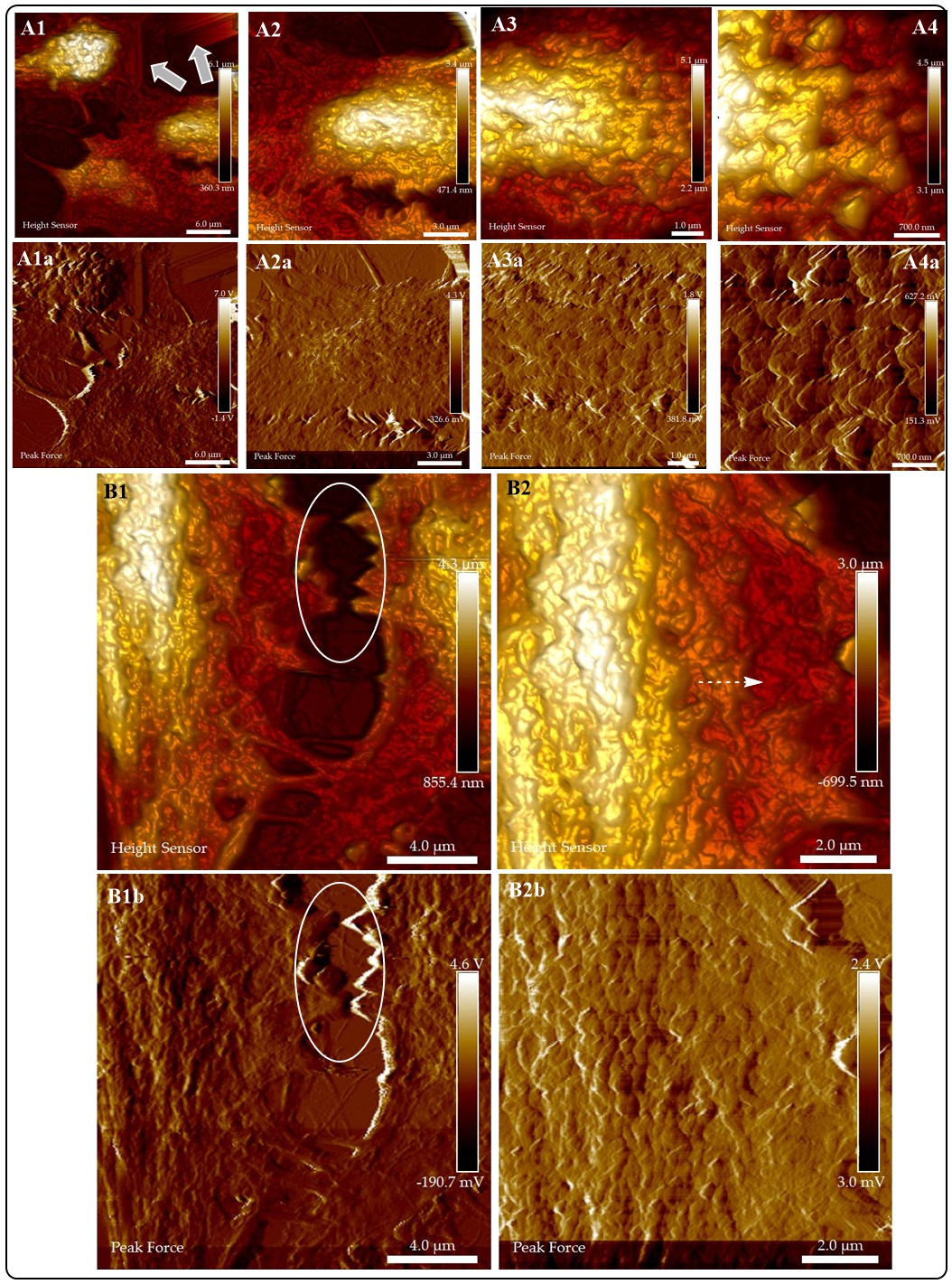
**

**Figure 12S.** AFM analysis of the interaction of SH-SY5Y cells and AZO 1% (5 µg/mL) NMs. A1-A4. SH-SY5Y cells exposed to 5 micrograms of AZO 1%. A1 Low magnification AFM images showing cells and cytoplasmic neurosecretory granules. A2-A4 High-magnification images showing the effect of AZO NMs in neurites (A2). A3, A4 AFM images demonstrate the presence of NMs in the surface of SH-SY5Y cells. B1-B2. AFM images showing damaged neurite cell connection.


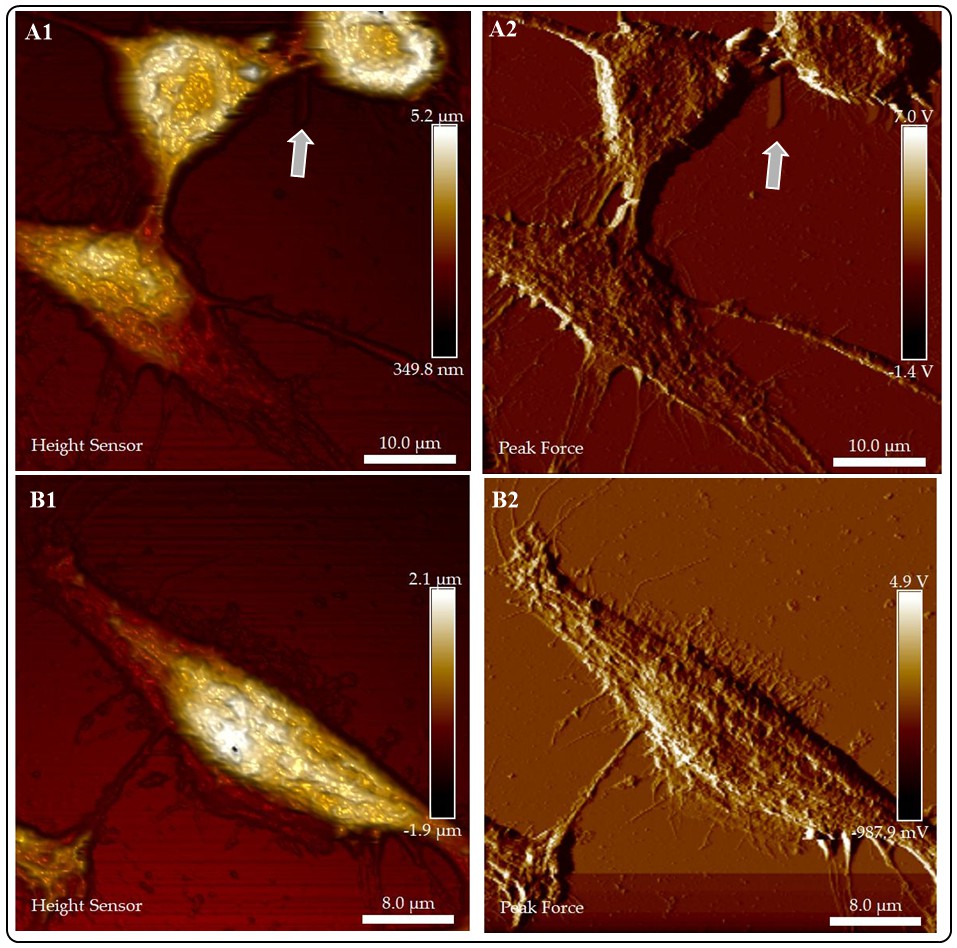


**Figure 13S.** AFM analysis of the interaction of SH-SY5Y cells and AZO 1 % (10 µg/mL). AFM images show different morphologies of SH-SYF% cells, cytoplasmic neurosecretory granules and neurite connection damage (white arrows).

**
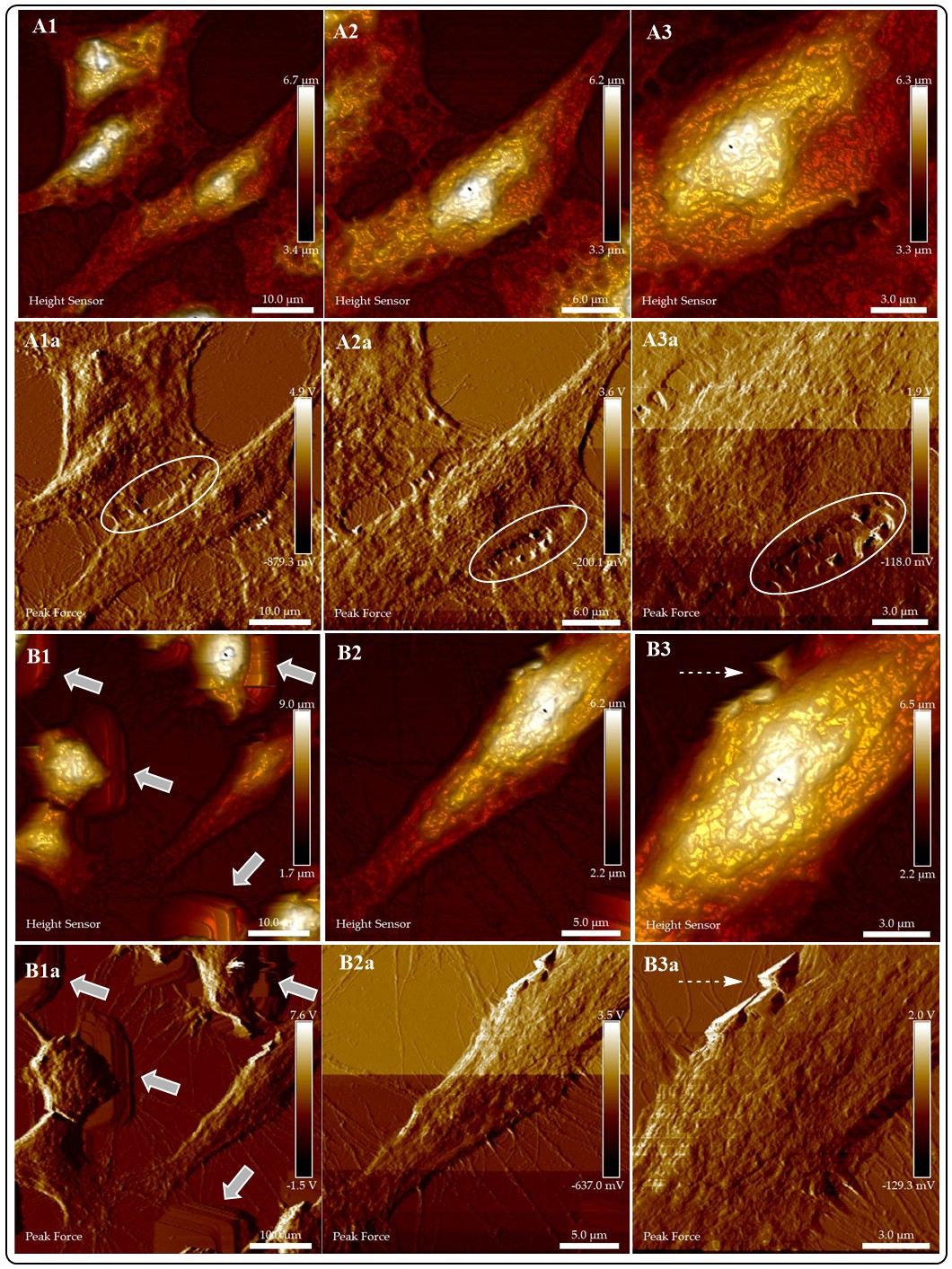
**

**Figure 14S.** AFM analysis of the interaction of SH-SY5Y cells and AZO 3% (5 µg/mL) NMs. AFM images show high resolution images of SH-SYF% cells, cytoplasmic neurosecretory granules (white arrows), membrane deformation (dotted arrow), and neurite connection damage (circles).

**
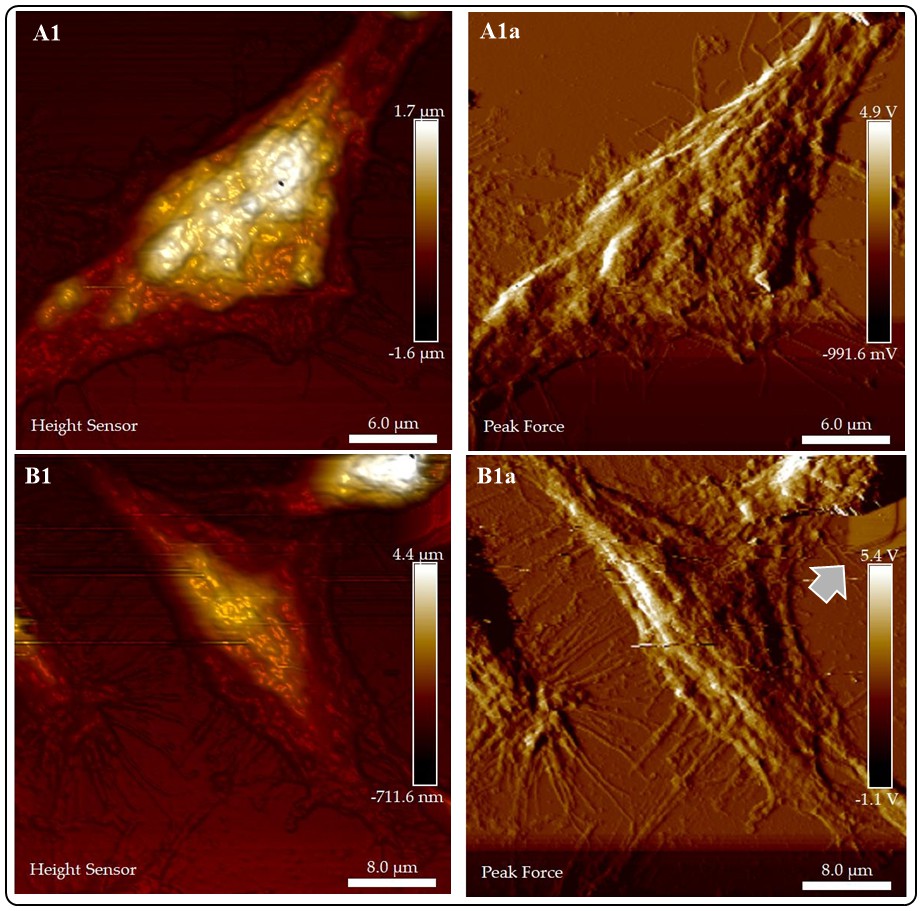
**

**Figure 15S.** AFM analysis of the interaction of SH-SY5Y cells and AZO 5% (5 µg/mL) NMs. AFM images show high resolution images of SH-SYF5Y cells, and cytoplasmic neurosecretory granules (arrow).

**
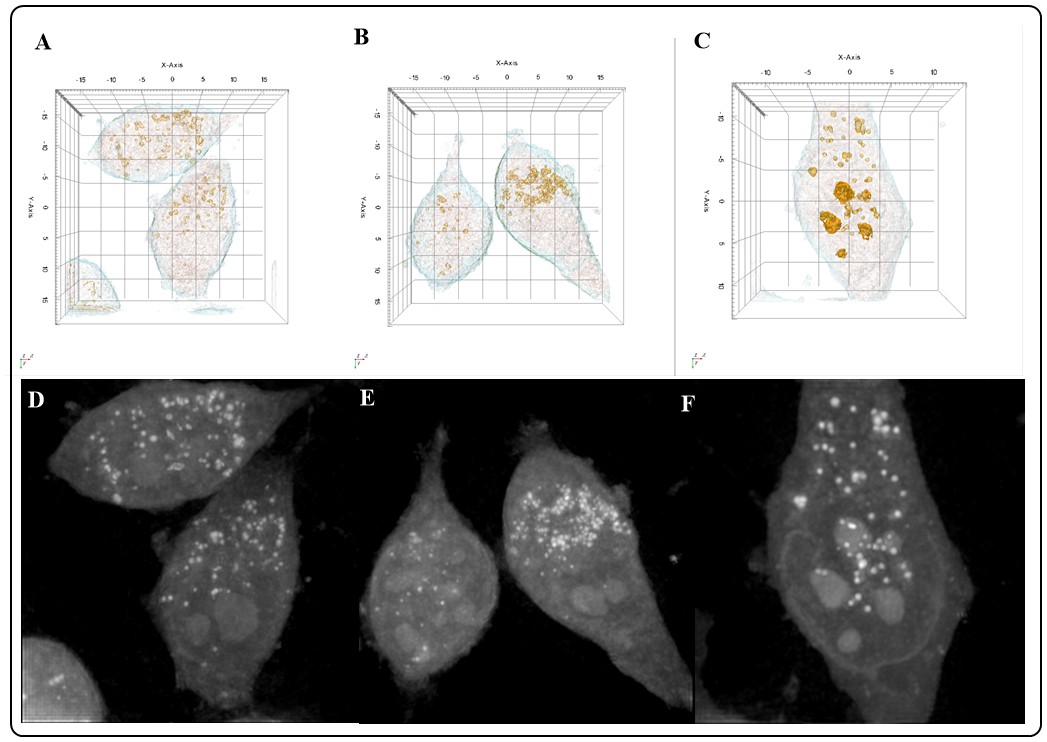
**

**Figure 16S.** HTM analysis of the interaction of SH-SY5Y cells with AZO NMs**.** Control group. **Top:** A,B,C: 3D representation of SH-SY5Y cells. **Bottom: D,E, F:** 2D optical phase projection image based on the theory of phase contrast microscopy.

**
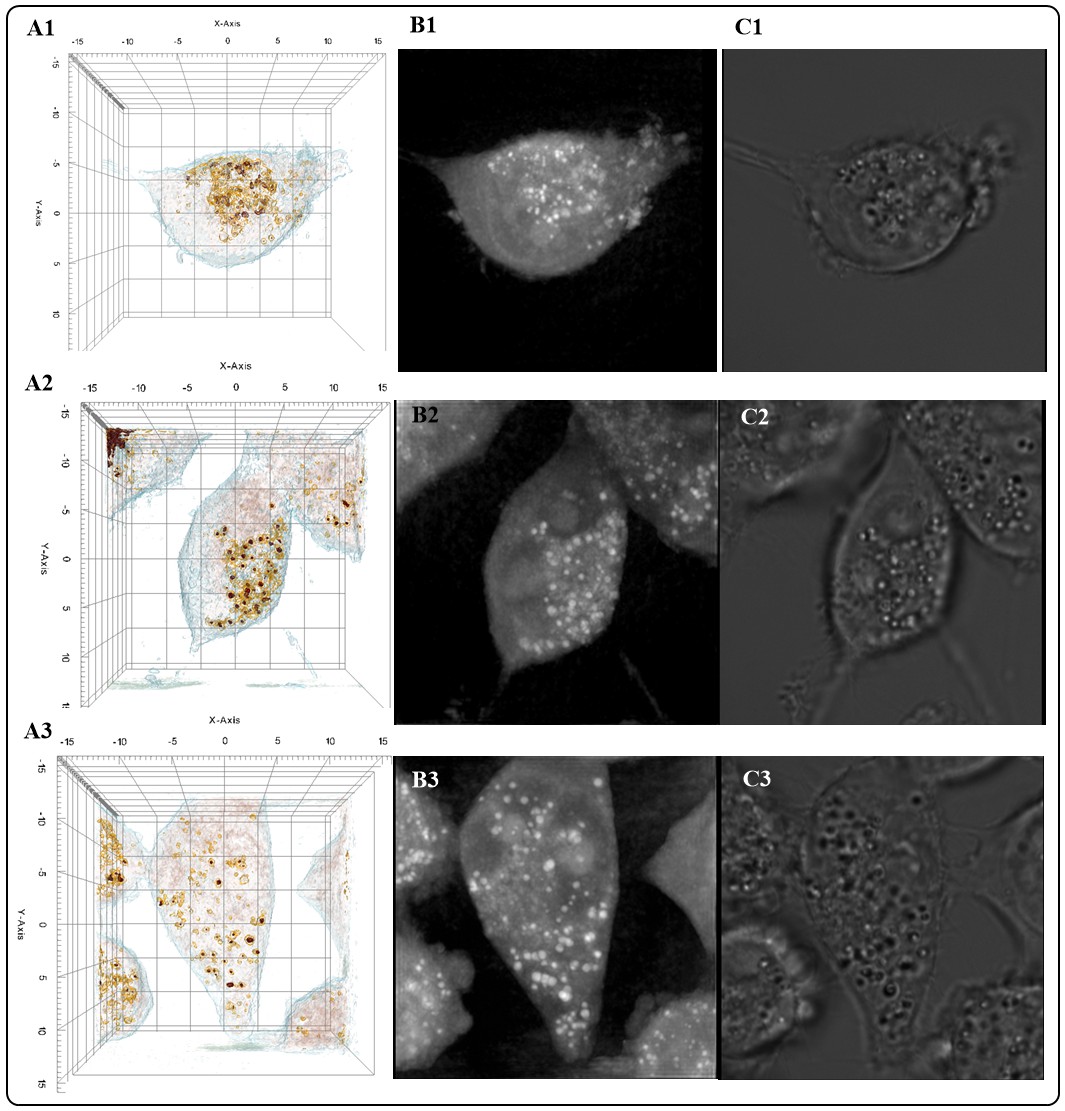
**

**Figure 17S.** HTM analysis of the interaction of SH-SY5Y cells with AZO 1% (5 µg/mL). **A** (1, 2, 3) 3D RI tomogram image colored according to RI values. **B** (1, 2, 3) 2D optical phase projection image based on the theory of phase contrast microscopy. **C** (1. 2. 3) Bright Field image. Red circles indicate the presence of Al:ZnO NMS inside the cells.

**
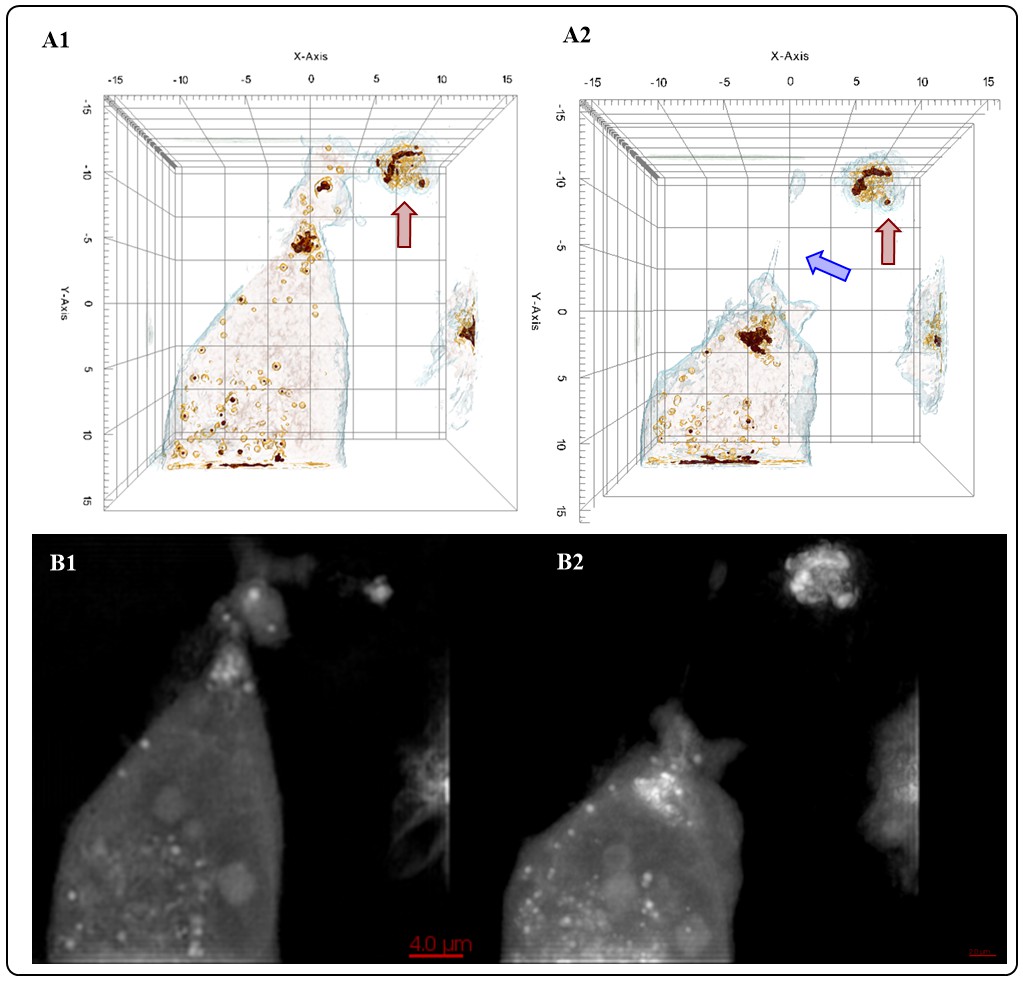
**

**Figure 18S.** HTM analysis of SH-SY5Y cells interacting with AZO NMs. A1) 3D representation of SH-SY5Y cell with AZO NMs (red dots) inside. Cytoplasmic neurosecretory granules (red arrow) encapsulate NMs and are secreted out of the cells. A2) SH-SY5Y cell excretes granule and moves away (blue arrow). B1)

| **NPs** | **Hydrodynamic size (nm)**  **/ PDI** | **Zeta potential (mV)** |
| --- | --- | --- |
| ZnO-Al^+3^ 1% | 228.73 ± 18.57 / 0.47 | 30.37 ± 2.65 |
| ZnO-Al^+3^ 3% | 218.77 ± 27.76 / 0.45 | 29.0 ± 2.32 |
| ZnO-Al^+3^ 5% | 200.27 ± 19.81 / 0.41 | 31.03 ± 0.81 |

**Table 1S.** Physicochemical properties of the different NMs in water
